# Adventitial Cell Atlas of wt (Wild Type) and ApoE (Apolipoprotein E)-Deficient Mice Defined by Single-Cell RNA Sequencing

**DOI:** 10.1161/ATVBAHA.119.312399

**Published:** 2019-04-04

**Authors:** Wenduo Gu, Zhichao Ni, Yuan-Qing Tan, Jiacheng Deng, Si-Jin Zhang, Zi-Chao Lv, Xiao-Jian Wang, Ting Chen, Zhongyi Zhang, Yanhua Hu, Zhi-Cheng Jing, Qingbo Xu

**Affiliations:** 1 From the School of Cardiovascular Medicine and Sciences, King’s College London British Heart Foundation Centre, United Kingdom (W.G., Z.N., J.D., Z.Z., Y.H., Q.X.); 2 Key Laboratory of Pulmonary Vascular Medicine and FuWai Hospital, State Key Laboratory of Cardiovascular Disease, Peking Union Medical College, Chinese Academy of Medical Sciences, Beijing (Y.-Q.T., S.-J.Z., Z.-C.L., X.-J.W., Z.-C.J.); 3 Department of Cardiology, The First Affiliated Hospital, Zhejiang University, China (T.C., Q.X.).

**Keywords:** adventitia, animals, atherosclerosis, mice, stem cells

## Abstract

**Objective—:**

Vascular adventitia encompasses progenitors and is getting recognized as the major site of inflammation in early stage of atherosclerosis. However, the cellular atlas of the heterogeneous adventitial cells, the intercellular communication, the cellular response of adventitia to hyperlipidemia, and its contribution to atherosclerosis have been elusive.

**Approach and Results—:**

Single-cell RNA sequencing was applied to wt (wild type) and ApoE (apolipoprotein E)-deficient aortic adventitia from 12-week-old C57BL/6J mice fed on normal laboratory diet with early stage of atherosclerosis. Unbiased clustering analysis revealed that the landscape of adventitial cells encompassed adventitial mesenchyme cells, immune cells (macrophages, T cells, and B cells), and some types of rare cells, for example, neuron, lymphatic endothelial cells, and innate lymphoid cells. Seurat clustering analysis singled out 6 nonimmune clusters with distinct transcriptomic profiles, in which there predominantly were stem/progenitor cell–like and proinflammatory population (Mesen II). In ApoE-deficient adventitia, resident macrophages were activated and related to increased myeloid cell infiltration in the adventitia. Cell communication analysis further elucidated enhanced interaction between a mesenchyme cluster and inflammatory macrophages in ApoE-deficient adventitia. In vitro transwell assay confirmed the proinflammatory role of SCA1^+^ (stem cell antigen 1 positive) Mesen II population with increased CCL2 (chemokine [C-C motif] ligand 2) secretion and thus increased capacity to attract immune cells in ApoE-deficient adventitia.

**Conclusions—:**

Cell atlas defined by single-cell RNA sequencing depicted the heterogeneous cellular landscape of the adventitia and uncovered several types of cell populations. Furthermore, resident cell interaction with immune cells appears crucial at the early stage of atherosclerosis.

## Abstract 2

Supplemental Digital Content is available in the text.

**Highlights**

- Single-cell RNA sequencing reveals the aortic adventitia as a dynamic interface harboring mesenchyme cells and immune cells, including T cells and macrophages.
- Adventitial nonimmune cells display significant heterogeneity of progenitor and fibroblast markers.
- A subpopulation of adventitial nonimmune cells expressing *Ly6a* attracts immune cells in ApoE (apolipoprotein E)^−/−^ mice.
- Ligand-receptor pair analysis predicted how resident mesenchyme cells interact and attract immune cells in vivo.

Atherosclerosis is characterized by vascular inflammation and represents a major mortality cause worldwide.^1^ Traditional inside-out theory of atherosclerotic lesion development encompasses macrophage adhesion on the luminal surface, endothelial dysfunction, leukocyte accumulation in subendothelial space, and subsequent inflammatory responses.^1^ These orchestrating mechanisms are established to initiate from the luminal side of the vessel. However, substantial evidence emerges and supports the outside-in theory.^2–4^ Events in the adventitia such as angiogenesis and inflammatory infiltration correlate with plaque development.^5–7^ Various cell types that reside in the dynamic adventitia including adventitial progenitors,^8^ myofibroblasts, and immune cells have been demonstrated to participate in vascular remodeling and contribute to atherosclerotic lesion development.^4,9,10^ It has been demonstrated that adventitia plays a key role in the development of neointima after vessel injury.^11–13^ However, approaches to define adventitial progenitors and immune cells that rely on limited and preselected markers do not necessarily reflect their in vivo diversity and heterogeneity.^8^ In addition, essential information about gene coordination is neglected because of the biased choices of genes to study. Adding another layer of complexity to the adventitial involvement in atherosclerosis, multiple cell types might interact and operate in concert to modulate lesion progress, and systemic study of cell communication has not been viable.

Single-cell RNA sequencing (scRNA-seq) offers an opportunity to unbiasedly interrogate thousands of genes simultaneously at an unprecedently high resolution. Depiction of adventitial cell landscape with scRNA-seq is imperative in characterizing the cellular heterogeneity, unraveling cellular identities, uncovering underlying disease-associated markers or cells, and shedding light on the potential cell communication mechanisms. Here, we performed scRNA-seq of aortic adventitial cells from wt (wild type) and ApoE (apolipoprotein E)-deficient (ApoE^−/^^−^) mice to explore their heterogenous identities, diverse functional states, dynamic cellular communications, and altered transcriptomic profiles in disease.

## Materials and Methods

The data that support the findings of this study are available from the corresponding author on reasonable request.

### Mice and Adventitial Cell Isolation

Twelve-week-old male wt and ApoE^−/−^ mice (C57BL/6J background, Jackson Laboratory) were fed on normal laboratory diet. To avoid data variation incurred by sex difference,^14^ only male mice were selected for the study. Mice were sacrificed with cervical dislocation. Perfusion was performed with 5 mL PBS through left ventricular puncture until the liver yields a pale color. Aorta (including aortic arch, thoracic aorta, and abdominal aorta) was pooled from 20 mice in each group (wt and ApoE^−/−^). Adventitia was carefully peeled off from the media and intimal layer for subsequent enzyme digestion. To obtain single cells, the pooled adventitia was washed with PBS 3× and then subjected with enzyme digestion with 5 mL 2 mg/mL collagenase I (Invitrogen; 17018–029) and 2 mg/mL dispase II (Sigma; D4693) in Hank balanced salt solution containing calcium and magnesium for 30 minutes. All procedures involving animals in the study follow the guidelines from Directive 2010/63/EU of the European Parliament on the protection of animals. Protocols from the Institutional Committee for Use and Care of Laboratory Animal and license issued by the UK Home Office were followed.

### Cell Sorting

Digested cells were filtered with 40 μm filter (Corning) and then centrifuged at 300*g* for 5 minutes. Cells resuspended in PBS were stained with LIVE/DEAD Fixable Near-IR (APC/Cy7 channel) Dead Cell Stain Kit (Invitrogen; L34975, 1:1000) and Hoechst 33342 (Invitrogen; H3570, 1:1000) for 20 minutes. Unstained cells and cells staining with only 1 fluorochrome prepared concomitantly served as control. After 1 wash in PBS for 5 minutes, cells were resuspended in PBS and then sorted with BD FACSARIA II. Nucleated live cells (Hoechst^+^/APC/Cy7^−^ population) were sorted into PBS with 0.04% BSA for subsequent scRNA-seq.

### Single-Cell RNA Sequencing

Standard 10x Chromium Single Cell 3′ v2 (10X Genomics GemCode Technology) protocols were followed for scRNA-seq. Briefly, single cells with specific 10x Barcode and unique molecular identifier were generated by partitioning the cells into Gel Bead-In-Emulsions. Subsequent cDNA sequences with the same 10x Barcode were considered as sequences from 1 cell. Library was generated and sequenced with Nova PE150. Sequencing depth was set to be 30 million per cell.

### Preprocessing of scRNA-seq Data

Raw sequencing data were demultiplexed, aligned, and counted with Cell Ranger pipelines. Basically, cellranger mkfastq command was used to generate fastq files, which were leveraged later by command cellranger count to produce expression data at a single-cell resolution. Cellranger aggr command combines sequencing data from multiple libraries with mapped sequencing depth.

### Clustering and Pathway Analysis of scRNA-seq Data

After aggregation of samples from wt and ApoE^−/−^ adventitial cells with mapped sequencing depth, R package Seurat was used for gene and cell filtration, normalization, principle component analysis, variable gene finding, clustering analysis, and t-distributed stochastic nearest neighbor embedding. Analyses were performed with default parameters unless otherwise specified. Briefly, matrix containing gene-by-cell expression data from the aggregated library were imported first to create a Seurat object. Cells expressing <200 or >2500 genes were filtered out for exclusion of noncell or cell aggregates. Cells with with a percentage of mitochondrial genes >0.05 were also filtered out. Data were then log-normalized for subsequent analysis. Principle component analysis was performed for dimension reduction. After calculation with JackStraw function, the first 10 principle components were used for clustering analysis. Clusters were visualized with t-distributed stochastic nearest neighbor embedding. Visualization of gene expression with violin plot, feature plot, dot plot, and heatmap was generated with Seurat function VlnPlot, FeaturePlot, DotPlot, and DoHeatmap, respectively. Markers for a specific cluster against all remaining cells were found with function FindAllMarkers (Arguments: only.pos=TRUE, min.pct=0.25). Differentially expressed genes (*P*<0.01) between 2 identities were found with FindMarkers function. Gene ontology (GO) and Kyoto encyclopedia of genes and genomes pathway analysis were performed with marker genes of each cluster found by FindAllMarkers function or enriched genes found by FindMarkers function with average log(fold change) >0.25 on DAVID (the database for annotation, visualization and integrated discovery) website and then plotted with R package ggplot2.

For subclustering of the nonimmune populations, raw data of these cells were retrieved from the Seurat object containing aggregated expression matrix for creation of a new and separate Seurat object. Similar gene filtration, principle component analysis, clustering, t-distributed stochastic nearest neighbor embedding, and pathway enrichment analysis were then performed. Cell cycle was analyzed by calculating the G1/S and G2/M scores, which were plotted in a 2-dimensional space as described.^15^ Briefly, the G1/S and G2/M scores were calculated by subtracting the mean expression value of the 10n nearest neighbors by expression level and detection frequency from the mean expression value of the n genes of the specified gene set.

### Weighted Gene Coexpression Network Analysis

Weighted gene coexpression network analysis (WGCNA) from R package WGCNA was used for identification of highly correlated gene modules. Briefly, adjacency matrix for signed cell correlation network was first created with a soft power set at 9 to allow for scale-free topology. Dissimilarity of topological overlap matrix was then used as input for hierarchical clustering of genes. Minimum number of genes included in each module were set to be 30. Total expression of genes within one module shown as verbose box plot was used to represent the module expression level. Gene correlation network within each module was visualized with R package igraph. Fruchterman-Reingold layout was applied and the size of the node correlated with the gene-module membership value of the corresponding gene. Module-trait relationship was calculated with Pearson correlation. For the correlation of modules with a specific cluster, the cluster being assessed was set to be 1, and the value of remaining clusters was set to be 0. Value of wt cells was set as 0 and value for ApoE^−/−^ cells was set as 1 for the analysis of module-trait (genotype: wt or ApoE^−/−^) correlation.

### Pseudotime Trajectory Analysis

Pseudotime trajectory analysis was performed with R package monocle (version 2.9) with default settings unless otherwise specified. Genes used for pseudotime ordering were taken from the first 1000 (by *P*) differentially expressed genes identified by function differentialGeneTest with fullModelFormulaStri set as cluster. DDRTree method was utilized for dimension reduction and cell ordering along the pseudotime trajectory. Branch analysis (branch points 1 and 2) was performed with branch expression analysis modeling. When presenting the significantly changed (*P*<0.01) genes in the branch point, 6 gene blocks were chosen according to the distinct patterns of gene expression change toward the 2 different cell states. Genes included in GO term cytokine activity or transcription factors (list obtained from transcription factor database^16^) were intersected with the 6 significantly changed gene blocks identified and presented as heatmap.

### Ligand-Receptor Cellular Communication Analysis

Ligand-receptor pairs were obtained from previously published data.^17^ In the analysis, transcriptomic level of ligands or receptors was taken for bioinformatic prediction of potential interactions at the protein level. After intersecting with genes detected, 2174 ligand-receptor pairs were kept (Table I in the online-only Data Supplement). When calculating ligand-receptor interactions, the ligand-receptor pair is counted if both the expression of ligand gene in the ligand cell and the expression of receptor gene in the receptor cell were above zero. Normalized expression data were used in the analysis. Mean number of ligand-receptor interaction between cell types was calculated by dividing the total number of ligand-receptor pairs (all ligand-receptor pairs were used in calculation) by the multiplication of ligand cell number and receptor cell number. The interaction of specific ligand-receptor pair between cell types was the total number of this ligand-receptor pair divided by the multiplication of ligand cell number and receptor cell number. Communication within selected cell types was demonstrated with chord graph generated by R package circlize. Color of the link depicted the ligand cell type. Percentage of cells expressing ligand gene (same color of link and cell type) or receptor gene (different color of link and cell type) was also shown in the graph. Same band color at both ends of the link illustrates interaction within this cell type. For a specific cell type (shown as the band of a specific color surrounding the circular graph), its total contribution to ligand-receptor interactions (ratio of its length to the total length of the band) and its contribution as ligand or receptor (ratio of the band length [same color as the link, ligand; different color from the link, receptor] to the total length of this color) could also be seen. Heatmap was generated with R package pheatmap.

### Culture of Adventitial Mesen II Cluster Cells

Adventitia progenitors were cultured in vitro as specified previously.^18^ Briefly, the adventitia explants from wt and ApoE^−/−^ mice were cultured on gelatin-coated flasks in stem cell medium (DMEM with 10% Embryomax, 10 ng/mL leukemia inhibitory factor, 0.1 mmol/L 2-mercaptoethanol, 100 U/mL penicillin, and 100 mg/mL streptomycin). Primary cells were sorted with anti–Sca-1 magnetic beads (Miltenyi Biotec) and a magnetic cell sorting system. Purified cells were passaged at 1:3 upon 80% confluence. Cells within 5 passages after sorting were utilized for subsequent studies.

### Quantitative Polymerase Chain Reaction

RNA was extracted with RNeasy mini kit (Qiagen) following standard protocols. QuantiTect Reverse Transcription Kit (Qiagen) was used for reverse transcription. Primers used were as follows: *Ccl2* (chemokine [C-C motif] ligand 2), forward, 5′-TTAAAAACCTGGATCGGAACCAA-3′; *Ccl2*, reverse, 5′-GCATTAGCTTCAGATTTACGGGT-3′. Fold change of gene of interest was calculated against internal control *GAPDH*. All samples were run in duplicates.

### Mouse CCL2 ELISA and Chemokine ELISA Array

Mouse CCL2 ELISA (R&D Systems; MJE00) and chemokine array (Qiagen; MEM- 009A) for supernatant of adventitial cells were performed with manufacturer’s protocol. First, 50 µL standard, control, or adventitial cell culture supernatant at different time points was added to ELISA microplates with 50 µL Assay Diluent and incubated for 2 hours at room temperature. After washing, 100 µL conjugates were added to each well and incubated at room temperature for 2 hours. One hundred microliters of stop solution was then added after incubation with 100 µL substrate solution for 30 minutes. Absorbance was read at 450 nm with wavelength correction at 540 nm within 15 minutes.

### Transwell Assay

Migration assays were performed using transwell inserts with 8.0 µm pore membrane filters (Corning). Bone marrow cells (10^5^ cells per 100 µL serum-free medium) were seeded into the upper chamber, whereas the bottom chamber contained vascular adventitial mesenchyme cell culture medium with or without CCL2-blocking antibody (R&D; AB-479-NA). Serum-free medium served as negative control. After 4 hours of incubation, nonmigrating cells on the upper side of the filters were carefully washed and removed using a swab. The migrated cells on the lower surface of transwell filter were fixed in 4% paraformaldehyde for 10 minutes and then stained with 1% crystal violet (Sigma; HT90132) for 15 minutes. Images were acquired using Nikon Eclipse TS100 microscope. Cells were counted in 5 random fields under the microscope for statistical analysis.

### En Face Immunofluorescent Staining of Adventitia

The staining protocol was modified from previous publication.^19^ Briefly, the adventitia from the thoracic aorta was separated from the media and intima before permeabilization with 0.2% Triton X-100 in PBS for 15 minutes, which was followed by blocking with 5% donkey serum at room temperature in Eppendorf tubes. Primary antibody was diluted 1:100 in PBS with 2% donkey serum over night at 4°C. Following antibodies were used: anti-LYVE1, ab14917 (Abcam); anti-PECAM1 (platelet and endothelial cell adhesion molecule 1), 553370 (BD Biosciences); anti-RBFOX3 (RNA-binding fox-1 homolog 3), ab177487 (Abcam); and anti-ACHE (acetylcholinesterase), MA3-042 (Life Technologies; please see the Major Resources Table in the online-only Data Supplement). After washing with PBS for 3× (5 minutes each), the adventitia was stained with secondary antibodies (Life Technologies; Alexa Fluor) diluted 1:500 in PBS. The nuclei were counterstained with DAPI (4′,6-diamidino-2-phenylindole). Stained tissue was mounted on slides with image taken with Leica SP5 confocal microscope.

### Availability of Data

The scRNA-seq data of wt and ApoE^−/−^ adventitia are available for reproducing the results. The authors declare that all R scripts used to process data are available from the corresponding author if requested.

### Statistical Analysis

Data with ≥5 experiment repeats passed Kolmogorov-Smirnov normality test that determines data normality and the *F* test that assesses homogeneity of variance. Unpaired and 2-tailed Student *t* test were applied to analyze data between 2 groups. Data were expressed as mean±SD using Graphpad Prism 6 software. Comparisons across multiple groups with 5 experiment repeats per group were assessed with 1-way ANOVA test, followed by Bonferroni post hoc analysis. Comparisons across multiple groups with 3 experiment repeats per group were assessed with Kruskal-Wallis test, followed by Bonferroni post hoc analysis. Experiment repeats in each group were specified in the figure legends. Appropriate significance was obtained with a relatively small group size. *P* <0.05 was considered statistically different.

## Results

### Depiction of Adventitial Cellular Landscape With scRNA-seq

**Figure 1.**
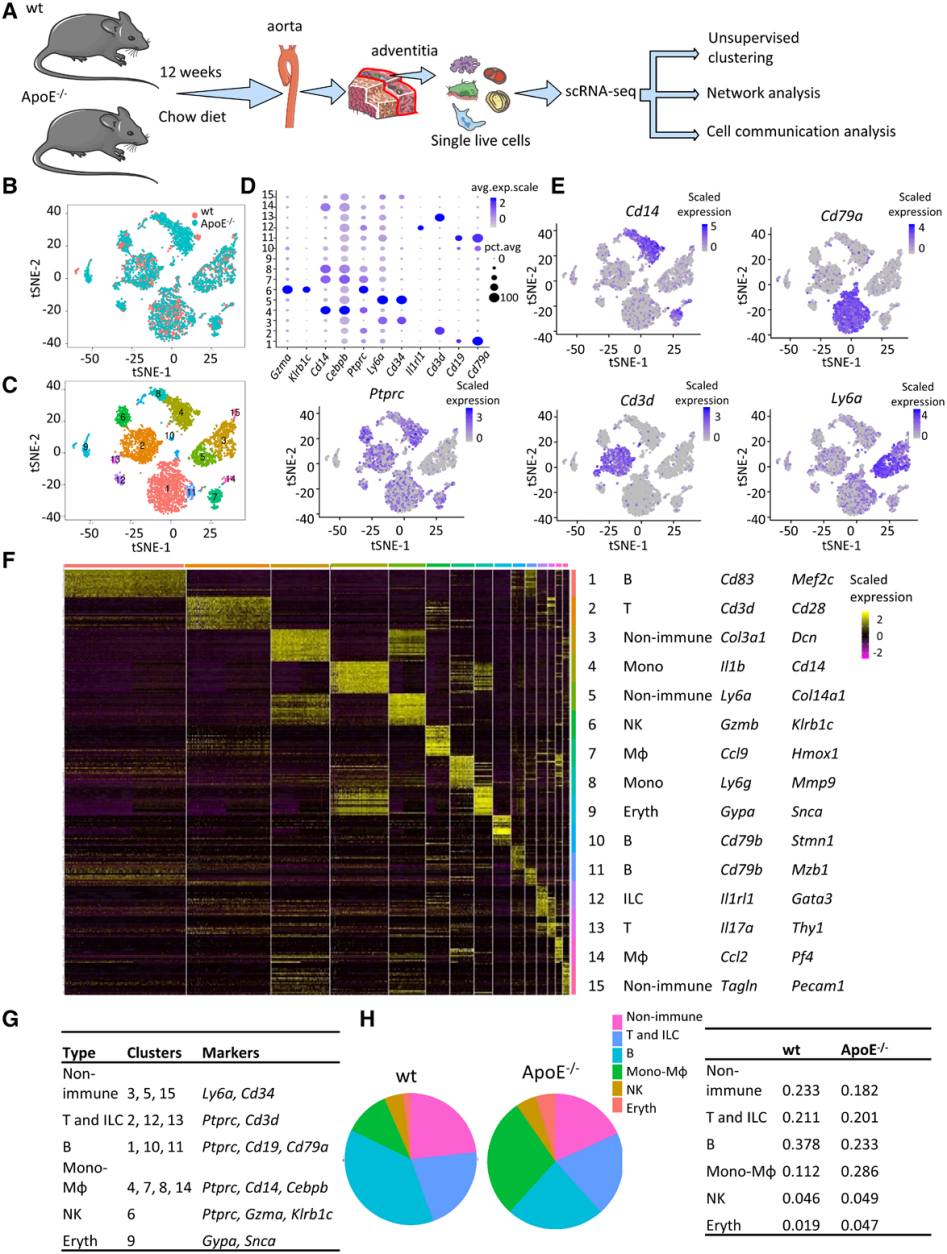
Identification of main cell clusters in the adventitia of male wt (wild type) and ApoE (apolipoprotein E)^−/−^ mice. **A**, Schematic graph of single-cell RNA sequencing and data analysis pipeline. **B**, t-distributed stochastic neighbor embedding (t-SNE) plot of adventitial cells from wt and ApoE^−/−^ mice. Colors denote different genotypes. **C**, t-SNE plot of adventitial cells with colors denoting cluster number. **D**, Dot plot of selected marker genes for each cluster. **E**, Feature plot of markers defining major cell types. **F**, Heatmap of the top 20 (by average log[fold change]) marker genes from each cluster and cell type assignment of each cluster. Full list of markers is in Table II in the online-only Data Supplement. **G**, Cluster and major cell type correspondence. **H**, Fraction of each cell type in wt and ApoE^−/−^ adventitial cells. Avg.exp.scale indicates average scaled expression; Eryth, erythrocyte; ILC, innate lymphoid cell; MΦ, macrophage; Mono, monocyte; NK, natural killer; pct.exp, percentage of expressing cells; and scRNA-seq, single-cell RNA sequencing.

In 12-week-old mice, the plasma cholesterol is significantly increased, and the aorta displayed small and sparse atherosclerosis lesions (Figure IA and IB in the online-only Data Supplement). Aorta is isolated after removal of perivascular fat (Figure IA in the online-only Data Supplement) and then peeled off with no soft connective tissue still attached to the media layer, as demonstrated by the nice colocalization of ACTA2 (actin alpha 2, smooth muscle) and DAPI in the media and endothelial layers (Figure IC and ID in the online-only Data Supplement). To characterize the adventitial cellular landscape, we obtained enzymatically dissociated adventitial cells from 12-week-old male wt and ApoE^−/−^ mice and sorted single nucleated live cells (Hoechst^+^/APC/Cy7^−^) for scRNA-seq (Figure 1A; Figure IE in the online-only Data Supplement). From wt and ApoE^−/−^ adventitia, 2271 and 3153 cells, respectively, were included in subsequent analysis after quality control. Similar mean reads per cell from wt and ApoE^−/−^ adventitia were achieved after aggregating 2 datasets with Cell Ranger to control for comparable sequencing depth (Figure IIA in the online-only Data Supplement). Data integration with canonical correlation allowed for alignment across conditions.^20^ Wt and ApoE^−/−^ cells displayed similar number of unique molecular identifiers, comparable number of genes, and aligned distribution along the canonical correlation subspace (Figure IIB in the online-only Data Supplement). In addition, *Apoe* expression in ApoE^−/−^ adventitia was significantly downregulated, confirming the genotype (Figure IIC in the online-only Data Supplement). Unbiased clustering performed with Seurat canonical correlation analysis identified 15 clusters as visualized with t-distributed stochastic nearest neighbor embedding (Figure 1B). Integrated wt and ApoE^−/−^ datasets displayed satisfactory alignment (Figure 1C) in the clustering analysis. In total, 15 clusters were singled out with top 20 (by average log[fold change]) markers for each cluster listed in Table II in the online-only Data Supplement. *Ptprc* (encoding pan-hematopoietic marker CD45 [cluster of differentiation 45]) was used to distinguish immune and nonimmune cells (Figure 1D and 1E). Major immune populations identified included the monocyte-macrophages (clusters 4, 7, 8, and 14), which featured the expression of *Cd14* and *Cebpb*; the B cells (clusters 1, 10, and 11), which demonstrated high expression of *Cd79a* and *Cd19*; and T cells (clusters 2, 12, and 13), which exhibited high expression of T-cell marker *Cd3d* (Figure 1D and 1E). Innate lymphoid cells (ILCs) encompass similar T-cell function and demonstrated high expression of *Il1rl1*^21^ and *Gata3* (Figure 1F). Other immune cells included natural killer cells with marker genes *Gzma*, *Gzmb*, and *Klrb1c* (Figure 1D and 1F). Of note, although expression of dendritic cell markers *Flt3*, *Zbtb36*, and *Itgax* was detected, no dendritic cluster was singled out, with natural killer cell marker *Gzma* highly expressed in the cluster showing the highest percentage of *Itgax* expression (Figure III in the online-only Data Supplement).

Nonimmune cells mainly included 3 clusters (clusters 3, 5, and 15). Cluster 3 and 5 cells both displayed high expression of ECM (extracellular matrix) proteins (*Col3a1* and *Col14a1*, respectively; Figure 1F). Cluster 5 cells also showed high level of stem cell marker *Ly6a* (encoding SCA1 [stem cell antigen 1]; Figure 1F). It was noteworthy that marker genes of cluster 3 and 5 cells displayed a heterogeneous bimodal expression pattern with extensive overlap of marker genes, which could be seen in the heatmap (Figure 1F). Cluster 9 seemed to be composed of erythrocytes/amyloid cells. Cluster 15 cells consisted mainly of vascular lineages including adventitial smooth muscle cells (SMCs; *Tagln*) and endothelial cells (*Pecam1*; Figure 1F). Collectively, distinct gene expression patterns across all clusters were observed (Figure 1F) with unbiasedly identified marker genes for each cluster listed in Table II in the online-only Data Supplement. Assignment of putative cell types to clusters was concluded in Figure 1G. Among the identified cell types, mesenchyme cells, T cells, and natural killer cells demonstrated similar fraction in wt and ApoE^−/−^ adventitia, whereas there was an increased fraction of monocyte-macrophages in ApoE^−/−^ adventitia and a resultant decrease of B-cell fraction (Figure 1H). Moreover, clustering of separate wt or ApoE^−/−^ dataset and integrated datasets yielded similar assignment of cells to major cell types identified (Figure IID in the online-only Data Supplement), suggesting the robustness of clustering results.

### Heterogeneity of Nonimmune Cells in the Adventitia

**Figure 2.**
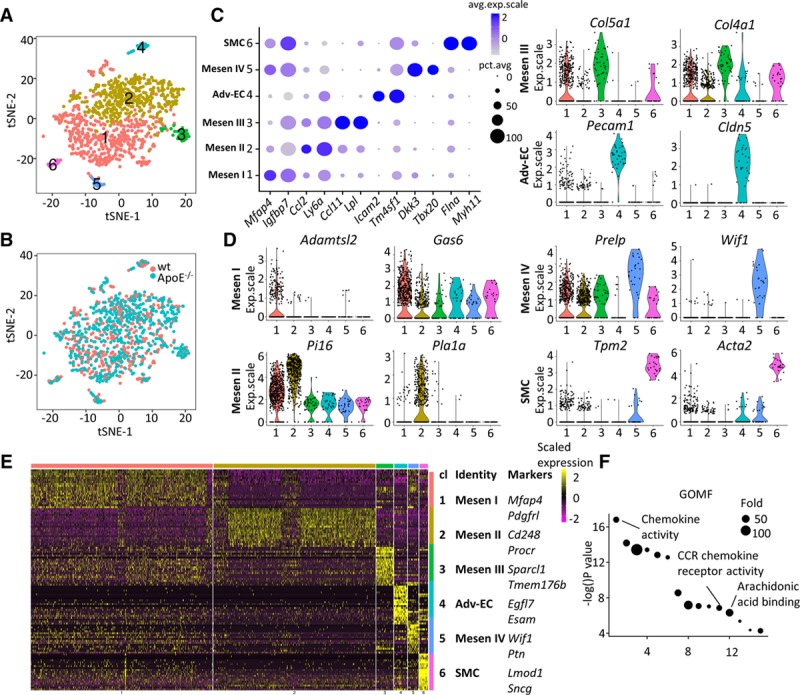
Clustering analysis of nonimmune cells from wt (wild type) and ApoE (apolipoprotein E)^−/−^ adventitia. **A** and **B**, t-distributed stochastic neighbor embedding (t-SNE) plot of adventitial nonimmune cells. Colors denote different clusters (**A**) or genotype (**B**). **C**, Dot plot of selected marker genes for each nonimmune cluster. **D**, Violin plot of marker genes of each nonimmune cluster. **E**, Heatmap of the top 20 (by average log[fold change]) marker genes from each nonimmune cluster and cell type assignment. Full list of markers is in Table III in the online-only Data Supplement. **F**, Gene ontology (GO) terms (molecular function) analysis of enriched (average log [fold change], >0.25) genes in ApoE^−/−^ Pecam1 (platelet/endothelial cell adhesion molecule 1)-positive endothelial cells in comparison with the corresponding wt cells. Adv-EC indicates adventitial endothelial cell; Avg.exp.scale, average scaled expression; CCR, C-C chemokine receptor; cl, cluster; exp.scale, scaled expression; GOMF, gene ontology molecular function; pct.exp, percentage of expressing cells; and SMC, smooth muscle cell.

After depicting the transcriptomic landscape of adventitia cells, we next sought to examine the nonimmune population. In previous clustering analysis, endothelial cells and SMCs were included in one cluster, whereas literature supports their distinct identities. Thus, clustering analysis of the nonimmune population was performed again with closer inspection to find markers of each subcluster relative to the rest of the nonimmune population. We aimed to infer the function of each subpopulation. Although adventitial mesenchyme cells received much attention in cardiovascular studies recently,^8^ scRNA-seq presents an opportunity to examine the adventitial cells unbiasedly without previous selection of marker genes. Seurat-based clustering analysis singled out 6 nonimmune clusters from the nonimmune population (clusters 3, 5, and 15), which were well aligned in wt and ApoE^−/−^ cells (Figure 2A and 2B). The marker genes for each cluster were listed in Table III in the online-only Data Supplement. In accordance with previous studies,^8,22^ considerable heterogeneity of stem cell markers, such as *Sca1*, *Cd34*, and *Tbx18*, and fibroblast markers, including *Ddr2*, *Col1a1*, and *Serpinh1*, was detected (Figure IVA and IVB in the online-only Data Supplement). Of note, cell proliferation did not serve as a heterogeneity source, as the proliferation markers including *Pcna*, *Mki67*, and *Mcm2* were not enriched in a specific cluster and the cell cycle analysis did not demonstrate significant difference among all the nonimmune clusters (Figure IVC and IVD in the online-only Data Supplement).

Among the 6 nonimmune clusters, Mesen I and Mesen II are the 2 major clusters, with significantly more cells than the remaining 4 (Figure 2A). Expression of endothelial markers *Pecam1* and *Cldn5*, adhesion molecules *Icam2* and *Esam*, endothelial cell–specific glycoprotein *Tm4sf1*, and endothelial angiogenic factor *Egfl7* allowed us to identify nonimmune cluster 4 as adventitial endothelial cells (Adv-ECs), possibly from the vasa vasorum (Figure 2C through 2E). Similarly, multiple genes (*Myh11*, *Flna*, *Tpm2*, and *Acta2*) specific for SMCs enabled the identification of nonimmune cluster 6 as SMCs (Figure 2C through 2E). In addition to adventitial SMCs, however, the medial SMC contamination could not be fully excluded, given that the adventitia was mechanically peeled off the aorta (Figure IE in the online-only Data Supplement). GO terms analysis was consistent with the cluster identities, with angiogenesis enriched in Adv-ECs and actin cytoskeleton organization enriched in SMCs (Figure V in the online-only Data Supplement).

After we confidently assigned the putative identities of cluster 4 and 6 to Adv-ECs and SMCs, we continued to explore the identities for the remaining mesenchyme clusters (Mesen I–IV) that were relatively elusive. Various ECM proteins (*Col15a1*, *Col4a1*, and *Sparcl1*) were enriched in Mesen III cluster (Figure 2D and 2E). Importantly, in Mesen III cluster, *Lpl*, which encoded the enzyme lipoprotein lipase, was enriched and *Ccl11* also displayed exclusive expression (Figure 2C). Among marker genes of Mesen IV cluster, *Dkk3* demonstrated importance in inducing smooth muscle differentiation,^23^ *Tbx20* was an essential transcription factor for cardiac development,^24^ *Prelp* was cartilage specific,^25^ and *Ptn* was a heparin-binding cytokine crucial for glial cell differentiation and angiogenesis^26^ (Figure 2C through 2E). Functional analysis of Mesen IV cluster marker genes also suggested diverse functions of the cells with enriched GO terms chondrocyte differentiation, negative regulation of ossification, and heart development (Figure V in the online-only Data Supplement).

Marker genes for Mesen I cluster included *Mfap4*^27^ and *Adamtsl2*^28^, ECM proteins important for elastic fiber and microfibril formation (Figure 2C and 2D). *Mfap4* accelerated neointima formation through promoting SMC migration,^29^ and a similar role of adventitial *Mfap4* might exist. In addition, *Gas6*, whose function in fibrotic diseases such as lung and liver fibrosis had been well characterized, was enriched in Mesen I cluster (Figure 2D).^30,31^ The enriched GO term ECM organization and collagen fibril organization further suggested the role of Mesen I cells in structural organization of adventitia (Figure V in the online-only Data Supplement). Tumor-suppressing genes *Igfbp7* and *Pdgfrl* were also highly expressed in Mesen I cluster (Figure 2C and 2E).^32,33^ For Mesen II cluster, stem cell marker *Ly6a*^18^ (encoding SCA1) and pericyte marker *Cd248*^34^ were enriched (Figure 2C through 2E). Interestingly, *Ccl2*—a chemokine secreted mainly by inflammatory cells and dysfunctional endothelial cells in atherosclerosis^35,36^—was selectively expressed in Mesen II cluster (Figure 2C). Other enriched genes in Mesen II cluster included *Pla1a*, which was activated in inflammatory conditions,^37^ and *Pi16*, which was regulated by shear stress and inflammation^38^ (Figure 2D). Moreover, apart from *Ccl2*, multiple other genes involved in inflammatory response were enriched in Mesen II cluster, including *Ccl7* and *Anxa1* (Figure VIA in the online-only Data Supplement). Consistently, GO term (biological function) analysis found enriched GO term cell adhesion with Mesen II marker genes (Figure V in the online-only Data Supplement).

After investigation of cluster identities, we next sought to examine the changes of adventitial cells in ApoE^−/−^ mice fed on normal laboratory diet in comparison with wt mice, which represented early stage of atherosclerosis. Given the different hemodynamics in the adventitial vasa vasorum and large arteries,^39^ it was hypothesized that adventitial vasa vasorum endothelial cells might also be dysfunctional in atherosclerosis development, similar to endothelial dysfunction of large arteries. To inspect this hypothesis, *Pecam1*-positive nonimmune adventitial cells from wt and ApoE^−/−^ mice were compared. GO terms analysis demonstrated enriched chemokine activity, CCR chemokine receptor activity, and arachidonic acid binding in ApoE^−/−^ *Pecam1* expressing adventitial cells (Figure 2F), showcasing early changes of Adv-ECs in atherosclerosis.

Collectively, we have identified 6 nonimmune clusters (Mesen I–IV, Adv-EC, and SMC) from the adventitial nonimmune population (clusters 3, 5, and 15). For convenience, markers for cluster identity interpretation mentioned above were summarized in a table (Figure VIB in the online-only Data Supplement). Confident identity assignment was achieved for Adv-EC and SMC clusters. Mesen III cluster was important in lipid metabolism according to the transcriptomic profile, and Mesen IV cluster displayed potential involvement in chondrocyte development, ossification, and heart development. Mesen I and Mesen II clusters, which were the 2 major clusters, included stem/progenitor cells that had a variety of potentials to differentiate into other cell types and demonstrated potential contribution to adventitia basal structure formation. To further characterize nonimmune clusters in the adventitia, we continued to explore the gene expression dynamics and cellular trajectories.

### Gene Correlation Dynamics of the Adventitial Nonimmune Population

Clustering analysis provided an opportunity to identify clusters and find marker genes of each cluster. However, the gene-gene correlation dynamics (relationship of genes) were neglected. To understand the gene expression dynamics, we used R package WGCNA, which utilized the dissimilarity topological overlap among genes to generate gene modules that contained correlated genes, which were regulated in a similar mode.^40^ The identified gene modules represented distinct cell identities^41^ or different cell states related to external traits.^42^

**Figure 3.**
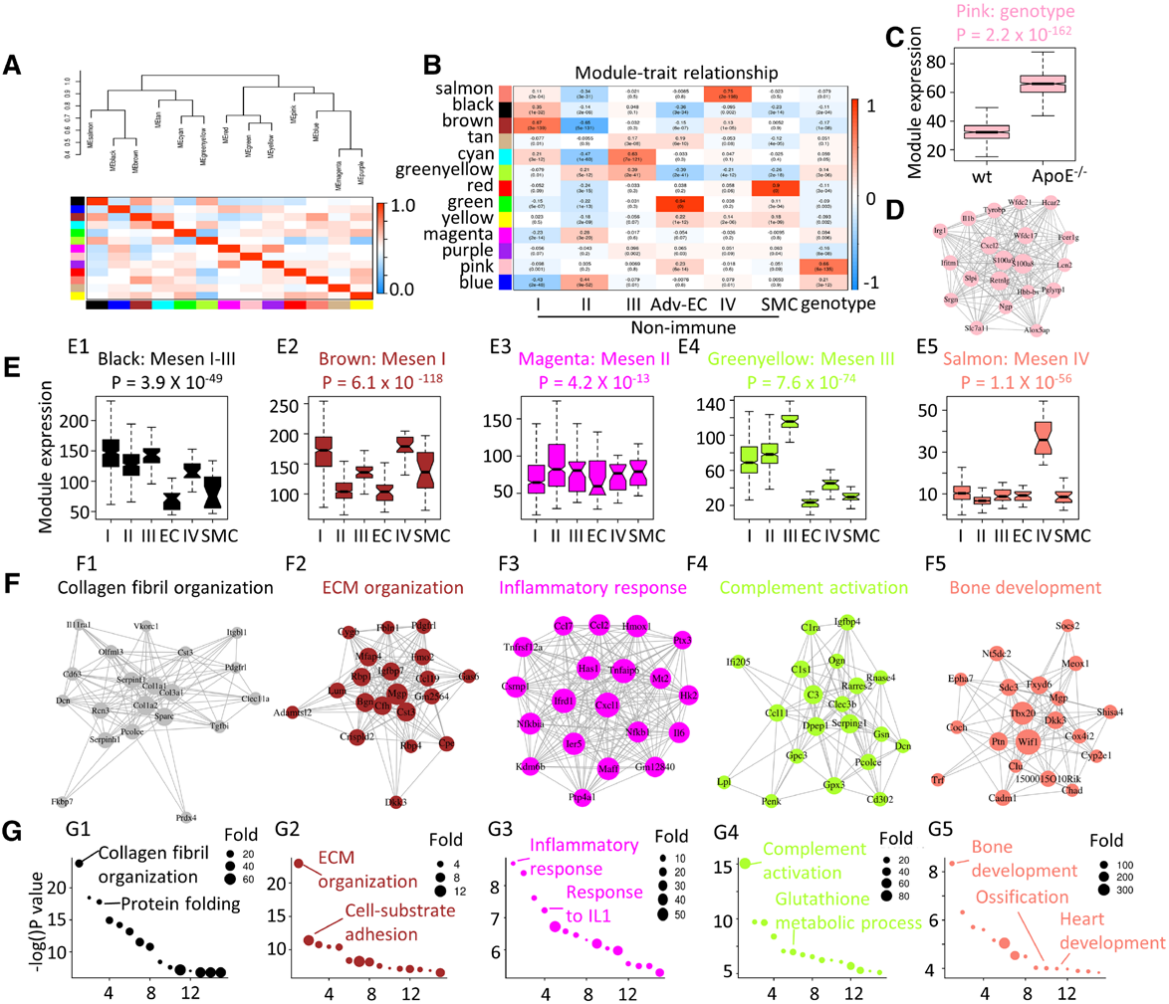
Gene expression dynamics of the nonimmune population. **A**, Eigengene network showing the clustering dendrogram with dissimilarity based on topological overlap and intercorrelation of each module identified by WGCNA. Color indicates modules. Color key indicates the correlation value. **B**, Correlation of gene modules with cell cluster identity (Mesen I–IV, adventitial endothelial cell [Adv-EC], and smooth muscle cell [SMC]) and genotype. Content in each cell represents the correlation value (first row) and the *P* value (second row). **C**, Gene expression distribution of genes from the pink module in wt (wild type) and ApoE (apolipoprotein E)^−/−^ mesenchyme cells were shown by box plot. **D**, Gene ontology (GO) terms (biological function) analysis of pink module genes. **E**, Gene expression distribution of module genes in each mesenchyme cell cluster (Mesen I–IV) was shown by box plot. **F**, Correlation network of the top 20 (by decreasing gene-module membership) genes in each module. Size of the node is in proportion to the gene-module membership, and the length of the link is in reverse correlation with the gene-gene correlation. **G**, GO terms (biological function) analysis of genes from each module. Colors indicate the module names. EC indicates endothelial cell; ECM, extracellular matrix; and IL1, interleukin 1.

In the adventitial nonimmune populations, we obtained 13 modules that contained genes to some extent correlated or changed in a similar manner (Figure 3A). Because of the large size (containing 1739 genes), the blue module might contain too many noise genes and was not included in downstream analysis. Correlation analysis of the gene modules with cluster identities revealed that the brown, magenta, cyan, green, salmon, and red modules were related to Mesen I, Mesen II, Mesen III, Adv-EC, Mesen IV, and SMC clusters, respectively (Figure 3B). The pink module highly associated with the genotype (wt or ApoE^−/−^; Figure 3B, last column), with upregulated expression of pink module genes in ApoE^−/−^ adventitial nonimmune cells (Figure 3C). As shown by the gene correlation network, *Cxcl2* and *Il1b*,^43,44^ 2 important cytokines in atherosclerosis were included in the pink module (Figure 3D). Interestingly, the correlation of gene modules with Mesen I and Mesen II clusters displayed a highly refined reverse trend, suggesting that these 2 clusters might be cells at different phases (Figure 3B, first and second columns). Consistent with previous cluster assignment, the green module that highly correlated with Adv-ECs contained mainly endothelial-specific genes and the red module that highly correlated with SMCs contained mainly smooth muscle–specific genes (Figure VII in the online-only Data Supplement). The greenyellow module was correlated with Mesen III cluster, and the most enriched GO term was complement activation (Figure 3B, 3E4, and 3F4). In Mesen IV cluster, associated salmon module displayed the highest expression (Figure 3E) and contained genes including *Tbx20*, *Dkk3*, and *Wif1* as illustrated in the network (Figure 3F5). In accordance with clustering analysis, these intercorrelated genes displayed similar enriched GO terms as the marker genes of Mesen IV cluster (Figure 3G5).

Although the black module was the most correlated with Mesen I cluster, it also displayed high expression in Mesen II and III clusters (Figure 3E1). According to the multiple enriched ECM genes (*Col1a1*, *Col3a1*, and *Col1a2*) in the center of the network and the enriched GO terms (collagen fibril organization and protein folding; Figure 3F1 and 3G1), it was inferred that Mesen I, Mesen II, and Mesen III clusters contributed to the ECM organization in the adventitia. The brown module genes showed high expression in Mesen I cluster and the hub gene (at the center of the network with the highest gene-module membership value) was *Mgp* (encoding matrix Gla protein)—a calcification inhibitor^45^ (Figure 3E2 and 3F2). Enriched GO terms in the brown module also included ECM organization, which was, in part, a confirmation of the brown and black module correlation (Figure 3A and 3G). Magenta module showed a modest high level in Mesen II cluster, and the genes in the correlation network included chemokines *Cxcl1* and *Ccl2*, which were included with the enriched GO term inflammatory response (Figure 3E3, 3F3, and 3G3).

To sum up, WGCNA correlation analysis allowed identification of modules containing functionally associated genes and demonstrated the transcriptomic dynamics. The 2 main Mesen clusters (by cell number, Mesen I and II) correlated with the brown and magenta module, respectively, enabling us to further the extend understanding of cluster identity and function. Supported by the most enriched GO terms, the modules were assigned ECM organization module and inflammatory response module (Figure 3F2, 3F3, 3G2, and 3G3).

### Pseudotime Trajectory Analysis of Adventitial Nonimmune Cells

**Figure 4.**
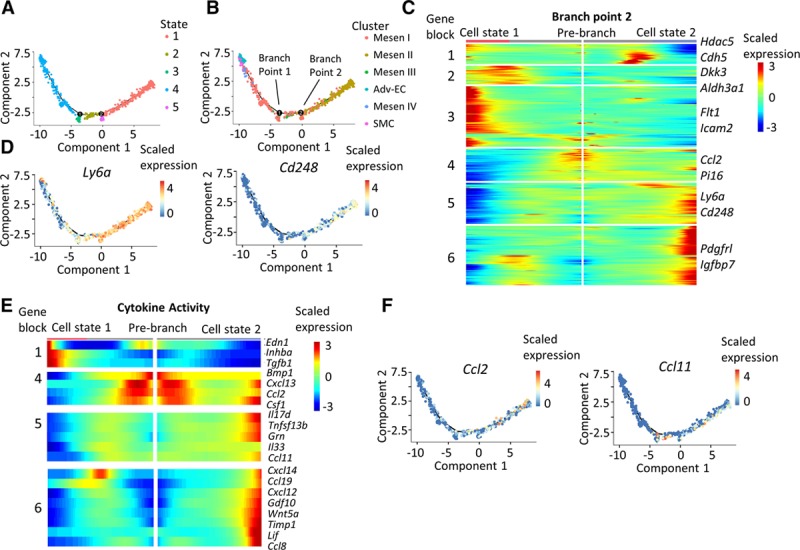
Pseudotime analysis discovers a proinflammatory role of Mesen II cluster. **A**, Distinct states of cells identified by pseudotime analysis. **B**, Ordering of cells from different nonimmune clusters along the pseudotime trajectory. **C** Heatmap of the significantly changed genes (*P*<0.01) discovered by the BEAM function from monocle in branch point 2. **D**, Expression level of *Ly6a* and *Cd248* along the pseudotime trajectory. **E**, Heatmap showing the expression level of significantly changed genes (*P*<0.01) in the gene ontology (GO) term cytokine activity. No significantly changed genes from cytokine activity GO term was found in gene blocks 2 and 3. **F**, Expression level of *Ccl2* and *Ccl11* along the pseudotime trajectory. Adv-EC indicates adventitial endothelial cell; BEAM, branch expression analysis modeling; and SMC, smooth muscle cell.

After investigating biological identities of the Mesen clusters and exploring the transcriptomic dynamics, we continued to inspect the relationships between different Mesen clusters. With pseudotime analysis, the nonimmune population was ordered along a trajectory, and cells at different states with 2 branching points were identified (Figure 4A). Adv-ECs, Mesen IV, and SMCs were found at one end of the trajectory, and Mesen II clusters were found at the other end (Figure 4B). Mesen I and Mesen III clusters lied in the middle of the trajectory (Figure 4B). Expression level of markers for different clusters further confirmed the cluster distribution along the pseudotime trajectory (Figure VIII in the online-only Data Supplement). Literature review presented some adventitia-derived cells as vascular stem/progenitor cells.^24^ Thus, we hypothesized that the branch point 1 represented to an extent the differentiation trajectory of adventitia progenitors to vascular lineages including Adv-ECs and SMCs. Analysis of branch point 1 discovered upregulation of both Adv-EC and SMC markers (*Pecam1*, *Acta2*, *etc*; Figure IX in the online-only Data Supplement). The upregulation of transcription factor *Erg*, which was essential for endothelial differentiation, further validated the utilization of branch point 1 to recapitulate vascular differentiation mechanism of stem/progenitor cells (Figure X in the online-only Data Supplement).^46^

Because analysis of branch point 1 to some extent resembled the differentiation mechanism, we next took advantage of branch point 2 analysis to gain insight about Mesen II cluster. First, analysis of branch point 2 uncovered upregulated gene blocks (gene block 3–6) toward cell state 2, with Mesen II marker genes *Ccl2*, *Pi16*, *Ly6a*, and *Cd248* in blocks 4 and 5 (Figure 4C). Plotting of *Ly6a* and *Cd248* along the pseudotime trajectory further demonstrated their upregulation when the cells were steered toward Mesen II cluster (Figure 4D). Interestingly, Mesen I marker genes *Pdgfrl* and *Igfbp7* were also in the upregulated gene blocks (block 6; Figure 4C). Furthermore, most genes involved in the GO term cytokine activity that were significantly changed in branch point 2 analysis were upregulated, among which were the proinflammatory cytokines *Ccl2* and *Ccl11*^47^ (Figure 4E and 4F).

Collectively, trajectory analysis unraveled the intercluster relationship of nonimmune subpopulations. At one end of the pseudotime trajectory were the mature cells including endothelial cells, SMCs, and Mesen IV cluster. The Mesen II cluster lied at the other end, implying a distinct role of it with other well-differentiated cells. Branching analysis revealed the upregulation of multiple chemokines while the cells were directed toward the inflammatory state.

### Transcriptomic Profile of Immune Populations in the Adventitia

**Figure 5.**
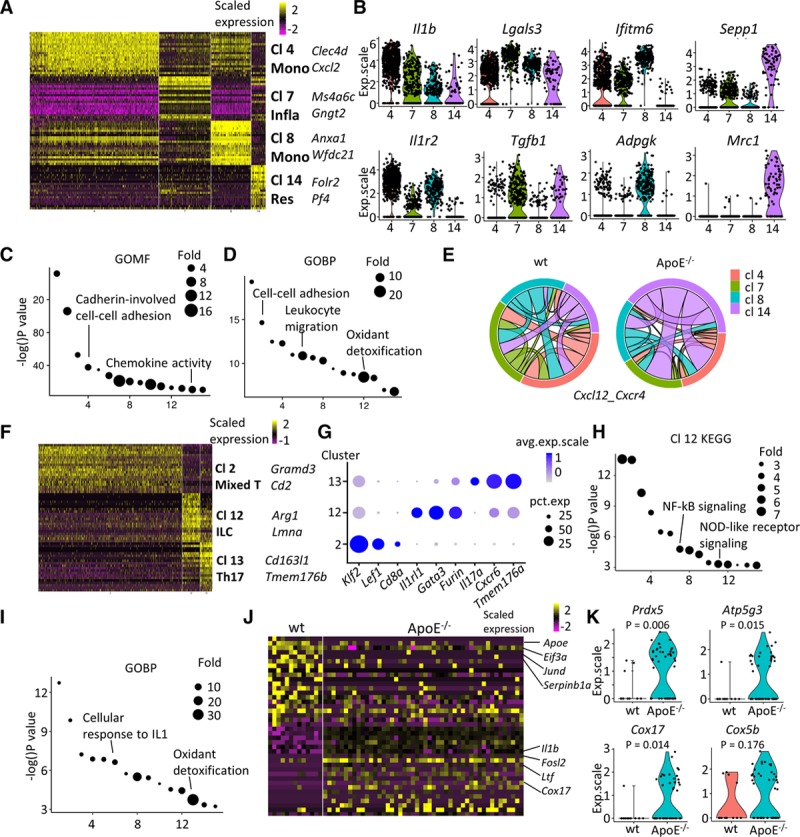
Characterization of immune cells from the adventitia. **A**–**D**, Monocyte-macrophages (clusters [Cl] 4, 7, 8, and 14). **A**, Heatmap of top 20 (by average log[fold change]) marker genes for each monocyte-macrophage Cl in comparison to the rest of the population. **B**, Violin plots of selected markers. **C**, Gene ontology (GO) term (molecular function) analysis of Cl 14 macrophages with its marker genes. **D**, GO term (biological pathway) analysis of genes significantly upregulated in Cl 14 resident–like ApoE (apolipoprotein E)^−/−^ macrophages in comparison to wt (wild type) analogy. **E**, Predicted interaction of *Cxcl12* and *Cxcr4* of wt and ApoE^−/−^ macrophages. The same color of link with the Cl indicates that cells from this Cl contribute to the interaction as ligand. Same band color at both ends of the link illustrates interaction within this cell type. **F**–**K**, T lymphocytes and innate lymphoid cells (Cl 2, 12, and 13). **F**, Heatmap of top 20 (by average log[fold change]) marker genes for each Cl relative to the rest of T lymphocytes. **G**, Dot plot of selected marker genes for each Cl of T lymphocytes. **H**, Kyoto encyclopedia of genes and genomes (KEGG) analysis of marker genes in Cl 12 innate lymphoid cells. **I**, GO terms (biological process) analysis of enriched (average log[fold change], >0.25) genes in ApoE^−/−^ Cl 12 innate lymphoid cells in comparison with the corresponding wt cells. **J**, Heatmap of top 20 (by decreasing *P*) enriched genes ApoE^−/−^ *Il1rl1*-positive T lymphocytes compared with corresponding wt cells. **K**, Violin plots of selected markers in ApoE^−/−^ *Il1rl1*-positive T lymphocytes compared with corresponding wt cells. ApoE indicates apolipoprotein E; exp.scale, scaled expression; GOBP, gene ontology biological pathway; GOMF, gene ontology molecular function; ILC, innate lymphoid cell; Infla, inflammatory macrophage; NF-κB, nuclear factor kappa B; NOD, nucleotide-binding oligomerization domain; and Res, resident-like macrophage.

After investigating the transcriptomic profiles of adventitial mesenchyme population, we next sought to unravel the transcriptomic heterogeneity of immune cells. The myeloid clusters (clusters 4, 7, 8, and 14) showed distinct gene expression patterns in comparison with the remaining myeloid populations (Figure 5A). Marker genes for cluster 4 included *Clec4d* and *Xcl2*, and for cluster 8, feature genes included *Anxa1* and *Wfdc21*. Cluster 4 monocytes demonstrated high expression of proatherosclerotic cytokine *Il1b* and its decoy receptor *Il1r2*,^48^ whereas cluster 8 monocytes showed high expression of *Adpgk* (encoding ADP-dependent glucokinase), which was important in glycolysis^49^ (Figure 5B). Marker genes for cluster 7 included *Ms4a6c* and *Gngt2* (Figure 5A), allowing us to identify it as inflammatory macrophages.^50^ Gene used for characterizing alternatively activated macrophage^51^ *Lgals3* was seen with the highest expression in cluster 7, further implying its inflammatory role (Figure 5B). Importantly, resident macrophage marker *Adgre1* (encoding *F4/80*) and aortic resident macrophage marker *F13a1* were enriched in both cluster 7 and cluster 14 (Figure XIA in the online-only Data Supplement). M2 macrophage markers *Folr2, Mrc1* (encoding CD206), and *Cbr2* were also exclusively expressed in cluster 14 (Figure 5A and 5B).^52^ The proatherosclerotic chemokines *Pf4*, *Sepp1*, and *C1qa* were also enriched in cluster 14 macrophages, consistent with previous reports^50^ (Figure 5A and 5B; Figure XIA in the online-only Data Supplement). Collectively, the evidence implied that cluster 7 was inflammatory macrophages and cluster 14 was resident macrophages.^50^ Indeed, GO term analysis revealed that cluster 14 marker genes participated in cadherin-involved cell-cell adhesion and chemokine activity signaling pathways (Figure 5C). Summary of markers used for cluster identification is shown in Figure XIB in the online-only Data Supplement. Next, comparison of wt and ApoE^−/−^ cluster 14 macrophages demonstrated that this cluster might play an important role in leukocyte attraction into the adventitia (Figure 5D). To inspect this, the communication of cluster 14 resident macrophages with other macrophage populations was examined. *Cxcl12*, which was an important anti-inflammatory cytokine, was found to have stronger interaction with other macrophage clusters in ApoE^−/−^ adventitia (Figure 5E), suggesting the significance of resident adventitial macrophages in the early stage of atherosclerotic lesion development.

The T cells and ILCs (clusters 2, 12, and 13) displayed distinct gene expression patterns (Figure 5F). In comparison with the remaining T cells and ILCs, cluster 2, cluster 12, and cluster 13 featured high expression of *Gramd3* and *Cd2*, *Arg1* and *Lmna*, and *Cd163l1* and *Tmem176b*, respectively (Figure 5F). Cluster 2 cells were identified as mixed T cells, with its high expression of specific T-cell surface marker *Cd8a* and the lymphocyte transcription factor *Lef1*,^53^ as well as transcription factor *Klf2* (Figure 5G). Cluster 13 cells expressed high level of *Il17a*—a specific cytokine for Th17 cells—in addition to *Cxcr6* and *Tmem176b* (Figure 5G). Cluster 12 cells showed high expression of *Il1rl1*—an ILC marker.^5^ In addition, cluster 12 cells exhibited an ILC2 (type-2 ILC) phenotype (*Cd3*^−^/*Il1rl1*^+^/*Thy1*^+^/*Il2ra*^+^)^54^ and showed expression of transcription factor *Gata3*, as well as some expression of type 2 cytokines (*Il5* and *Il13*; Figure XIIA in the online-only Data Supplement). Kyoto encyclopedia of genes and genomes analysis of cluster 12 ILC2 cells revealed that the enriched Kyoto encyclopedia of genes and genomes terms included NF-κB (nuclear factor kappa B) signaling and NOD (nucleotide-binding oligomerization domain)-like receptor signaling, which were essential in innate immunity (Figure 5H), further suggestive of the ILC identity.^55,56^ The markers used for cluster identification are summarized in Figure XIIB in the online-only Data Supplement. To further examine whether the potential role of ILCs in the early onset of atherosclerosis, comparison of their gene expression profile between wt and ApoE^−/−^ mice was performed. As exhibited by the GO terms (biological function) analysis, cluster 12 ILC2 cells showed upregulation of genes involved in cellular response to IL1 and oxidant detoxification (Figure 5I), which represented the early changes of ILCs in ApoE^−/−^ adventitia. Detailed characterization of wt and ApoE^−/−^ *Il1rl1*-positive ILC2 cells verified the gene expression profile changes in ILC2 population, including the downregulated *Eif3a*, *Jund*, and *Serpinb1a*, as well as the upregulated *Il1b*, *Fosl2*, *Ltf*, and *Cox17* (Figure 5J). Of note, gene *Prdx5* encoding an antioxidant enzyme and multiple genes related to mitochondrial respiration *(Atp5g3*, *Cox17*, and *Cox5b*) were upregulated in ApoE^−/−^ *Il1rl1*-positive ILC cells (Figure 5K; Figure XIII in the online-only Data Supplement).

### Mesen II Interaction With Adventitial Macrophages

**Figure 6.**
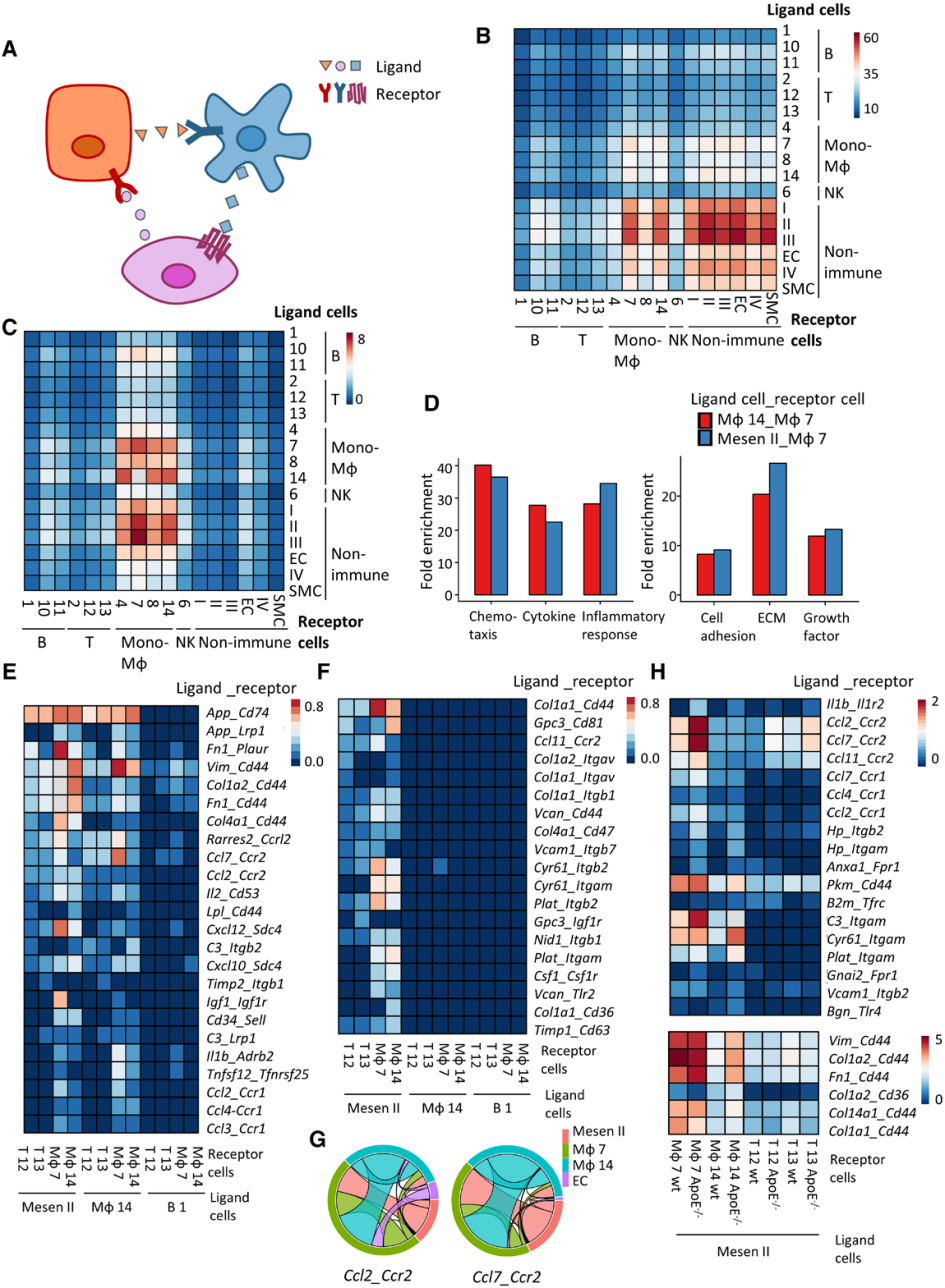
Mesen II cells interact with immune cells. **A**, Illustration of cell-cell interaction analysis. Ligand is from one cell (same color as the cell) and interacts with receptor from another cell. **B**, Mean interaction numbers between cell types from ApoE (apolipoprotein E)^−/−^ adventitia. Rows represent ligand cells, and columns represent receptor cells. **C**, Mean interaction numbers of ligands and receptors from the gene ontology inflammatory response gene set between cell types from ApoE^−/−^ adventitia. Rows represent ligand cells, and columns represent receptor cells. **D**, Gene set enrichment analysis (UniProtKB key words) of the ligands from the top 200 (mean number of interaction) ligand-receptor pairs of ligand cell type (resident MΦ 14 and Mesen II) and receptor cell type (inflammatory MΦ 7). **E**, Heatmap of mean interaction numbers of specified ligand-receptor pairs between specified cell types from ApoE^−/−^ adventitia. **F**, Heatmap of mean interaction numbers of specified ligand-receptor pairs between specified cell types from ApoE^−/−^ adventitia. **G**, Interaction of *Ccl2* and its receptor *Ccr2*, *Ccl7* and its receptor *Ccr2* between ApoE^−/−^ Mesen II, inflammatory macrophages (MΦ 7), resident macrophages (MΦ 14), and adventitial endothelial cells (ECs) from ApoE^−/−^ adventitia. The same color of link with the cluster indicates that cells from this cluster contribute to the interaction as ligand. **H**, Heatmap showing the comparison of mean interaction numbers of specified ligand-receptor pairs between Mesen II and specified cell types from wt (wild type) and ApoE^−/−^ adventitia. ECM indicates extracellular matrix; Mesen, mesenchyme; NK, natural killer; and SMC, smooth muscle cell.

Leveraging scRNA-seq, intercellular communication between heterogenous populations has been revealed to shape organ development.^57^ The complex interaction of various adventitial cell types and their mediation of atherosclerosis development was evaluated in our study by examination of the transcriptomic level of ligands and corresponding receptors (Figure 6A). Of note, the interactions in our study are computationally predicted rather than biological. In the adventitia, intercellular communication within mesenchyme populations (Mesen I–IV) and between mesenchyme populations and the monocyte-macrophages (Mono-MΦ clusters 4, 7, 8, and 14) were the dominating interactions, suggesting the importance of mesenchyme populations in maintaining adventitial homeostasis (Figure 6B; Figure XIVA in the online-only Data Supplement). Furthermore, their communication with monocyte-macrophages demonstrated stronger intercellular cross talk in ApoE^−/−^ adventitia (Figure XIVB in the online-only Data Supplement). Particularly, cells expressing *Cd34* and *Cav1* interacted with *Sell* and *Icam1* expressed by inflammatory macrophages (MΦ 7), respectively, which may potentially modulate leukocyte influx to the adventitia (Figure XV in the online-only Data Supplement).^58,59^

Further dissection of the interactions revealed that ligand-receptor pairs included in the GO term inflammatory response contributed to the communication between mesenchyme populations (Mesen I–III) and monocyte-macrophages to a similar extent as its contribution to cellular cross talk within monocyte-macrophages (Figure 6C; Figure XVIA in the online-only Data Supplement). Importantly, stronger cellular interaction reflected by the mean interaction numbers was also observed in ApoE^−/−^ adventitia (Figure XVIB in the online-only Data Supplement). Of note, the cellular interaction calculation robustness was confirmed by the stable interaction pattern when the cell number effect was adjusted (Figure XVIC and XVID in the online-only Data Supplement). Comparable enrichment of chemotaxis, cytokine, and inflammatory response between the interaction of Mesen II with inflammatory macrophages (MΦ 7) and interaction of Mesen II with resident macrophages (MΦ 14) further indicated the role of Mesen II in leukocyte chemotaxis (Figure 6D). Specificity of the comparable enrichment was confirmed by lower enrichment of inflammatory response between the interaction of Mesen II and Adv-ECs (Figure XVII in the online-only Data Supplement). Mesen II and MΦ 14 exhibited a similar mode in interaction with inflammatory cells in various ligand-receptor pairs that are involved in inflammatory response, including *Ccl2-Ccr2* (ligand-receptor) and *Ccl7-Ccr7* (Figure 6E and 6G; Figure XVIII in the online-only Data Supplement). As expected, the ligands from Mesen II cells that pair with inflammatory macrophages (MΦ 7) demonstrated higher enrichment in ECM in comparison with ligands that pair with resident macrophages (MΦ 14; Figure 6D).

Ligand-receptor pairs that selectively existed in Mesen II interaction with immune cells mainly involved those with matrix protein as ligands including *Col1a1-Cd44*, *Col1a2-Itgav*, and *Timp1-Cd36* (Figure 6F). Ligand-receptor pairs such as *Ccl24-Ccr2* and *C1qa-Lrp1* selectively existed in the interaction of resident macrophages and inflammatory macrophages, suggesting the inflammatory role of resident macrophages (Figure XIX in the online-only Data Supplement). Increased interaction of various ligand-receptor pairs including *Ccl2-Ccr2*, *Ccl7-Ccr2*, and *Il1b-Il1r2* between Mesen II and inflammatory cells in ApoE^−/−^ adventitia further implied the participation of Mesen II in early development of atherosclerosis (Figure 6H). Interestingly, various interactions involving matrix proteins as ligand were also increased in Mesen II and inflammatory cell interactions (Figure 6H). Overall, our exploration of intercellular communications suggested a proinflammatory role of Mesen II cells through interaction with inflammatory cells, particularly inflammatory macrophages, implying the participation of Mesen II in initiating adventitial inflammation in response to elevated blood lipid levels at the early stage of atherosclerosis. Additionally, the top 50 ligand-receptor pairs between all cell types are shown in Table IV in the online-only Data Supplement, which might offer insights for researchers interested in further studying intercellular communication.

### CCL2 Secreted by Adventitial Mesen II Cells Attracts Immune Cells

**Figure 7.**
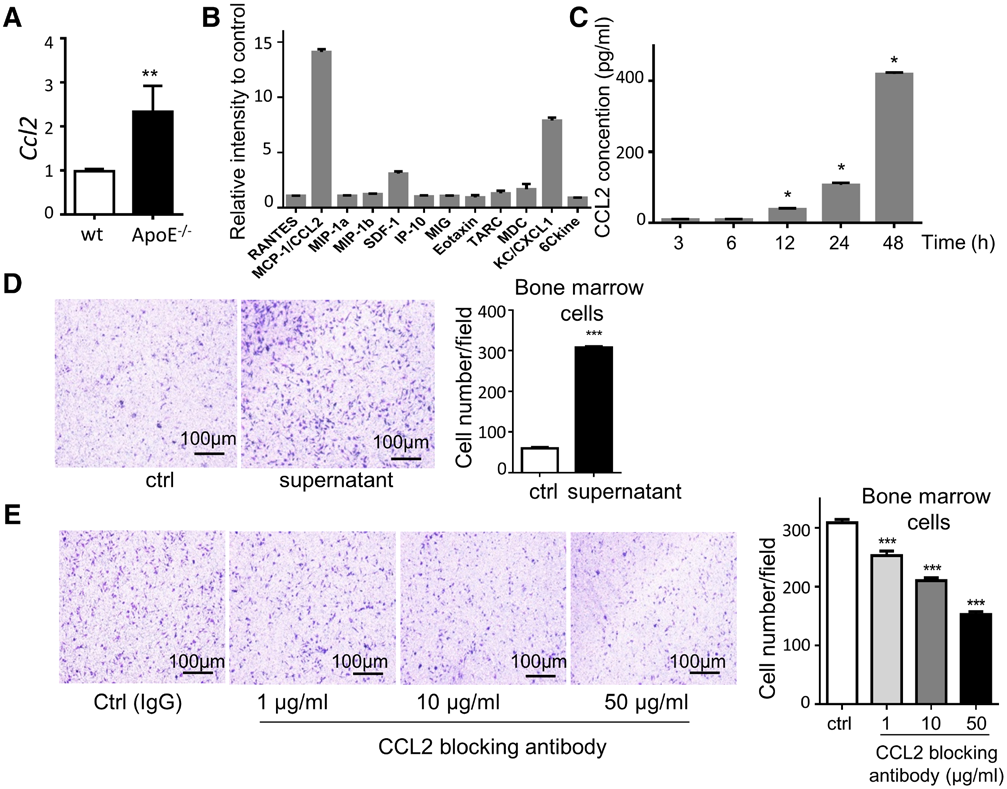
Proinflammatory role of stromal cells in the adventitia. **A**, Gene expression of *Ccl2* in in vitro cultured Sca-1+ adventitial mesenchyme cells with Gapdh mRNA level as internal control. n=5. **B**, Chemokine array of in vitro cultured Sca-1+ adventitia mesenchyme cells. n=3. **C**, Protein level of CCL2 in cell culture supernatant detected by ELISA. Time indicates the time for cells in culture. n=3. Significance determined with Kruskal-Wallis test in comparison with the previous time point was shown. **D**, Representative image and correspondent analysis of the migration assay (4 h) showing the attraction of bone marrow cells by ctrl (serum-free medium) and cell culture supernatant (18 h). n=5. **E**, Representative image and correspondent analysis of the migration assay (4 h) showing the attraction of bone marrow cells by cell culture supernatant (18 h) with IgG control or indicated concentrations of CCL2-blocking antibody. n=5. Significance is determined with 1-way ANOVA test. ApoE indicates apolipoprotein E; CCL2, chemokine (C-C motif) ligand 2; SCA-1, stem cell antigen-1; and wt, wild type. *P<0.05, **P<0.01, ***P<0.001.

After unraveling the proinflammatory role of Mesen II cluster of adventitial mesenchyme cells, experiments were then performed in vitro to investigate their role in attracting immune cells as a proof-of-concept validation. Sca-1 (encoded by *Ly6a*) was a frequently used marker for adventitial mesenchyme cells^8^ and highly expressed in Mesen II cluster (Figure 2C). Thus, Sca-1-positive cells were selectively enriched for separation of Mesen II adventitial cells from the nonimmune population. An increasing trend of *Ccl2* was detected in the ApoE^−/−^ adventitial cells (Figure 7A). Additionally, in the supernatant of adventitial Sca-1^+^ cells, CCL2 displayed the highest level among other detected chemokines and also a time-dependent upregulation (Figure 7B and 7C). Furthermore, supernatant from adventitial (Mesen II) cell culture media induced bone marrow cell migration, which was attenuated by the CCL2-blocking peptide (Figure 7D and 7E). Taken together, CCL2 was secreted by adventitial Sca-1^+^ cells and functioned in vitro as a chemoattractant for bone marrow cells. This proof-of-concept study aided to establish the proinflammatory role of adventitial Mesen II cluster.

### Rare Cell Types Detected by scRNA-seq

**Figure 8.**
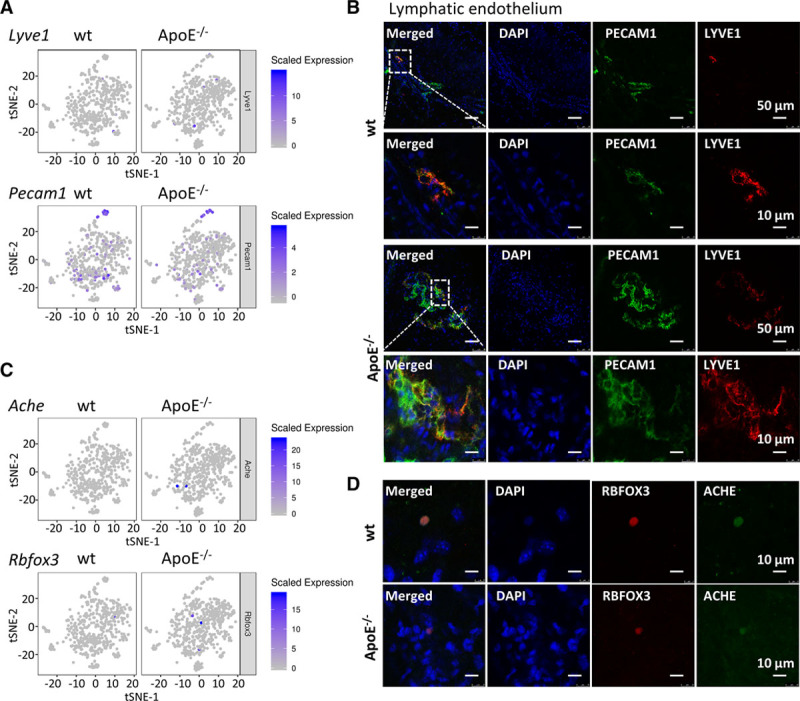
Rare cell types detected by single-cell RNA sequencing of the adventitia. **A**, Feature plot of lymphatic endothelium markers *Pecam1* and *Lyve1* in wt (wild type) and ApoE (apolipoprotein E)^−/−^ adventitial mesenchyme cells. **B**, En face staining of wt and ApoE^−/−^ aortic adventitia of PECAM1 and LYVE1. **C**, Feature plot of neuronal markers *Rbfox3* and *Ache* in wt and ApoE^−/−^ adventitial mesenchyme cells. **D**, En face staining of wt and ApoE^−/−^ aortic adventitia for RBFOX3 and ACHE. ACHE indicates acetylcholinesterase; ApoE, apolipoprotein E; DAPI, 4′,6-diamidino-2-phenylindole; LYVE1, lymphatic vessel endothelial hyaluronan receptor 1; PECAM1, platelet and endothelial cell adhesion molecule 1; RBFOX3, RNA-binding fox-1 homolog 3; and t-SNE, t-distributed stochastic neighbor embedding.

In the end, existence of rare cell types in the adventitia was checked. Adventitial lymphatics played a crucial role in the transport of cholesterol from the vessel wall to the blood stream and correlated with the plaque development in the intima.^60^ Here, in our study, *Lyve1* (lymphatic vessel endothelial hyaluronan receptor 1) expressing cells were detected in both wt and ApoE^−/−^ mesenchyme population, although because of their paucity, no distinct cluster was singled out (Figure 8A; cluster identity is indicated in Figure 2A). At the protein level, LYVE1^+^/PECAM1^+^ lymphatics were seen in the en face staining of adventitia (Figure 8B). Negative control stained with IgG controls displayed no positive staining (Figure XX in the online-only Data Supplement). In addition, neuronal markers including *Rbfox3* (encoding neuronal nuclei) and *Ache* (encoding achetylcholinesterase) were detected in the adventitial mesenchyme population (Figure 8C; cluster identity is indicated in Figure 2A). *Rbfox3* is a specific neuronal maker, and *Ache* degrades acetylcholine in cholinergic synapses and is involved in hypertension.^61,62^ The existence of neurons in the adventitia was further confirmed with immunostaining of RBFOX3 and ACHE (Figure 8D). Although these rare cells were not identified as separate clusters, validation of their existence in the adventitia could broaden our understanding of adventitia function in atherosclerosis.

## Discussion

With scRNA-seq, we unbiasedly depicted the cellular landscape of aortic adventitia, characterized resident and bone marrow–derived cell populations, and displayed several rare types of cells, including neurons, ILCs, and lymphatic endothelial cells. First, we found that a cluster of resident mesenchyme cells expressing stem/progenitor markers could be a source of several maturely differentiated cells, for example, endothelial and SMCs. Second, one subpopulation of adventitial mesenchyme cells demonstrated a proinflammatory role, with the function to attract immune cells to the adventitia through increased interaction of CCL2 and its receptors in ApoE^−/−^ mice. Third, resident macrophages in the adventitia seem to be activated at the early stage of hyperlipidemia. Finally, ligand-receptor pair analysis predicted how resident mesenchyme cells interact and attract immune cells in vivo. Thus, the information of adventitial cell atlas provided by scRNA-seq could be useful for understanding the roles of a variety of cells in atherogenesis in response to hyperlipidemia.

Examination of the mesenchyme populations in the adventitia unveiled heterogeneity of previously appreciated cell types including Sca-1^+^, CD34^+^, Tbx20^+^ stem cell marker–positive cells,^63^ and Ddr^+^ and Thy1^+^ fibroblast marker–positive cells.^64^ Additionally, heterogeneous adventitial progenitors and fibroblasts seem to display overlap to an extent that prompts caution when interpreting lineage-tracing studies exploring function of these cells because the markers selected might only label subpopulation of the adventitial cells.^64^ It is noteworthy that enriched GO terms including bone development and ossification suggested a possible role of Mesen IV cluster in vascular calcification, and adventitial cells were found involved in lesion calcification.^65^ Intriguing high expression of *Tbx20* in Mesen IV cluster also proposed the involvement of this cluster in cardiomyocyte differentiation. Spontaneous differentiation of CD34-positive adventitial cells toward cardiomyocyte was reported.^66^ Taken together, adventitia harboring stem/progenitor cells have a potential to produce several types of mature cells contributing to vascular remodeling and disease development.

For the 2 main clusters of the adventitial mesenchyme population, Mesen I and Mesen II, ECM proteins were highly expressed, backing their fundamental function in forming the basic adventitial structure. Furthermore, the WGCNA-identified modules that contained intercorrelated genes demonstrated reverse correlation with Mesen I and Mesen II cluster, supporting the hypothesis that Mesen I and Mesen II possibly represented 2 phases of 1 cell type. Similar to the macrophage polarization theory, a proinflammatory role of Mesen II population was proposed, which was further supported by the monocle-generated pseudotime trajectory. Validation of this proinflammatory role of Mesen II cluster was achieved by confirming the attraction of bone marrow cells in a CCL2-dependent manner. Our study has implied that intercellular communication alterations were early events in atherosclerosis development.

In terms of immune cells, previous studies have established their extensive heterogeneity in advanced plaques and revealed the proinflammatory role of nonfoamy macrophages.^50,67,68^ Restricting view to the adventitia, the 4 identified monocyte-macrophage clusters included 2 macrophage populations: inflammatory macrophage cluster and resident macrophage cluster. Resident population expressed proatherogenic chemokine *Pf4*, consistent with scRNA-seq data from all immune cells of atherosclerotic aorta.^50^ In atherosclerosis-prone ApoE^−/−^ adventitia, altered pathways in resident macrophages included cell-cell adhesion and leukocyte migration, indicating its underlying role in priming adventitia inflammation. With the characterized transcriptomic profile, resident macrophages might be involved in the cell activation in response to hyperlipidemia, which attracts immune cells. In fact, in the adventitia of ApoE^−/−^ mice, increased percentage of monocyte-macrophages was observed in comparison with the wt adventitia.

Tertiary lymphoid organs encompassing T cells, B cells, and other types of immune cells were reported to emerge in nonresolving inflammations in aortic adventitia, particularly in the abdominal aorta.^69^ Consistently, multiple lymphocyte populations were uncovered in our study. Interestingly, apart from the mixed T cells and Th17 cells, an *Il1rl1*-positive cluster was discovered, which fit the ILC identity. Importantly, this population did not express pan-T-cell marker *Cd3d* (Figure 1B), basophil marker *Mcpt8*,^57^ or mast cell marker *Enpp1* (encoding CD203c)^70^ (data not shown). ILCs mirror the T-cell function and represent a novel avenue in immunology.^21^ Protective role of type-2 ILCs in perivascular adipose tissue implied that this population in the adventitia, which was located in more proximity to atherosclerotic lesions, might undertake important function in regulating lesion development.^71^ Moreover, increased expression of genes related to oxidative phosphorylation in *Il1rl1*-positive population in adventitia from ApoE^−/−^ mice fed on normal laboratory diet demonstrated the early modulation of this cell type in atherosclerosis. Function of this previously unrecognized cell type from aortic adventitia in atherosclerosis merits further investigations. As to dendritic cells, no separate cluster was found in our study, possibly suggesting the involvement of dendritic cells in later stages of atherosclerosis as suggested by previous studies.^4,6^

Adding another layer of diversity to the cellular landscape of adventitia, lymphatic endothelial cells and neuronal cells were detected by scRNA-seq despite their rarity. Adventitial lymphatics reversely transport cholesterol and correlate with intimal thickness and thus atherosclerosis progression.^60,72^ Although sensory nerves (including cholinergic nerves characterized by ACHE expression) have been reported to exist in the adventitia, assisted with scRNA-seq, our study detected RBFOX3-positive neurons in the adventitia,^73^ which may be important in vessel contraction.

Altogether, utilization of ApoE^−/−^ mice fed on normal laboratory diet enabled us to characterize the fine-tuned interaction between cells in the adventitia in the early stage of atherosclerosis and uncover early landscape shift of adventitial cells. Interference of these early events bears the potential to prevent or reverse atherosclerotic lesions.

In the end, limitations of our study exist in the following aspects. The first limitation lies in the restricted inclusion of samples. Because our attention was mainly cast on adventitial cells during early stage of atherosclerosis development, only wt and ApoE^−/−^ adventitia from mice fed on normal laboratory diet was examined. Based on primary results from this study, we aim to sequence adventitial cells from other atherosclerotic models (LDLR [low-density lipoprotein receptor]^−/−^ mice), mice fed on Western diet, aging mice, and mice with advanced atherosclerotic lesions fed with statin in the future. Second, although the number of cells analyzed is sufficient in supporting the analysis in this study, future sequencing of more cells or selective enrichment of a specific population would provide further insight in rare populations in the adventitia. Third, we focused on analyzing the sequencing data in depth, and the in vitro experiments served mainly as a proof-of-concept study. Based on the wealth of information provided by the sequencing data, however, extensive in vitro experiments could be performed in the future to selectively enrich specific clusters and validate their role (such as ILCs) during atherosclerotic lesion development. Lastly, although we intend to gain a whole view of the adventitial cell atlas without preselection of subclusters, enzymatical dissociation of single cells induced damage more in branched and large cells and less in small cells like lymphocytes, which might introduce systematic bias in the study and lead to underrepresentation of macrophages. In situ RNA sequencing carries the potential to solve this issue.^74^ Future research also includes establishment of function in each cell type in atherosclerosis using lineage-tracing models with cluster-specific markers.

In summary, adventitia is gradually acknowledged as an essential interface harboring diverse cell types among which both mesenchyme cells and inflammatory cells exist and participate in vascular disease progression. With scRNA-seq, we managed to systematically characterize the cellular landscape of the dynamic adventitia at a single-cell resolution, present interesting populations to study and illustrate a proinflammatory subpopulation of adventitial mesenchyme cells, which served as a proof-of-concept study for their involvement in the early development of atherosclerosis.

## Acknowledgments

We thank Yaqin Zhang for the sketches of adventitia isolation process.

## Sources of Funding

This work is supported by the British Heart Foundation (RG/14/6/31144), National Science Fund for Distinguished Young Scholars (81425002), Chinese Academy of Medical Sciences Innovation Fund for Medical Sciences (2016-I2M-1-002, 2017-I2M-B&R-02, and 2016-I2M-4-003), National Natural Science Foundation of China (81630003), and Beijing Natural Science Foundation (7181009).

## Disclosures

None.

## Supplementary Material
