# Supplementary material for: Adventitial Cell Atlas of wt (Wild Type) and ApoE (Apolipoprotein E)-Deficient Mice Defined by Single-Cell RNA Sequencing
Source: Arterioscler Thromb Vasc Biol. 2019 Apr 4;39(6):1055–71. doi: 10.1161/ATVBAHA.119.312399 (PMC6553510; doi:10.1161/ATVBAHA.119.312399)
Supplement: Supplementary file 1 [file atv-39-1055-s001.pdf]

SUPPLEMENTAL MATERIAL

Adventitial Cell Atlas of Wild-type and ApoE-deficient Mice Defined by Single-cell RNA-sequencing

Wenduo Gu, Zhichao Ni, Yuan-Qing Tan, Jiacheng Deng, Si-Jin Zhang, Zi-Chao Lv, Xiao-Jian Wang, Ting Chen, Zhongyi Zhang, Yanhua Hu, Zhi-Cheng Jing, and Qingbo Xu

Correspondence to

Qingbo Xu, MD, PhD, School of Cardiovascular Medicine & Sciences, King's College London BHF Centre, 125 Coldharbour Lane, London SE5 9NU, United Kingdom Telephone: (+44) 20 7848-5322, Fax: (+44) 20 7848-5296, Email: qingbo.xu@kcl.ac.uk

or Zhi-cheng Jing, MD, Key Lab of Pulmonary Vascular Medicine & FuWai Hospital, State Key Laboratory of Cardiovascular Disease, Peking Union Medical College and Chinese Academy Medical Sciences, No. 167 Beilishi Road, Beijing, 100037, China, Tel: (+86)10-88396018, Fax: (+86)10-88396016, Email: jingzhicheng@vip.163.com

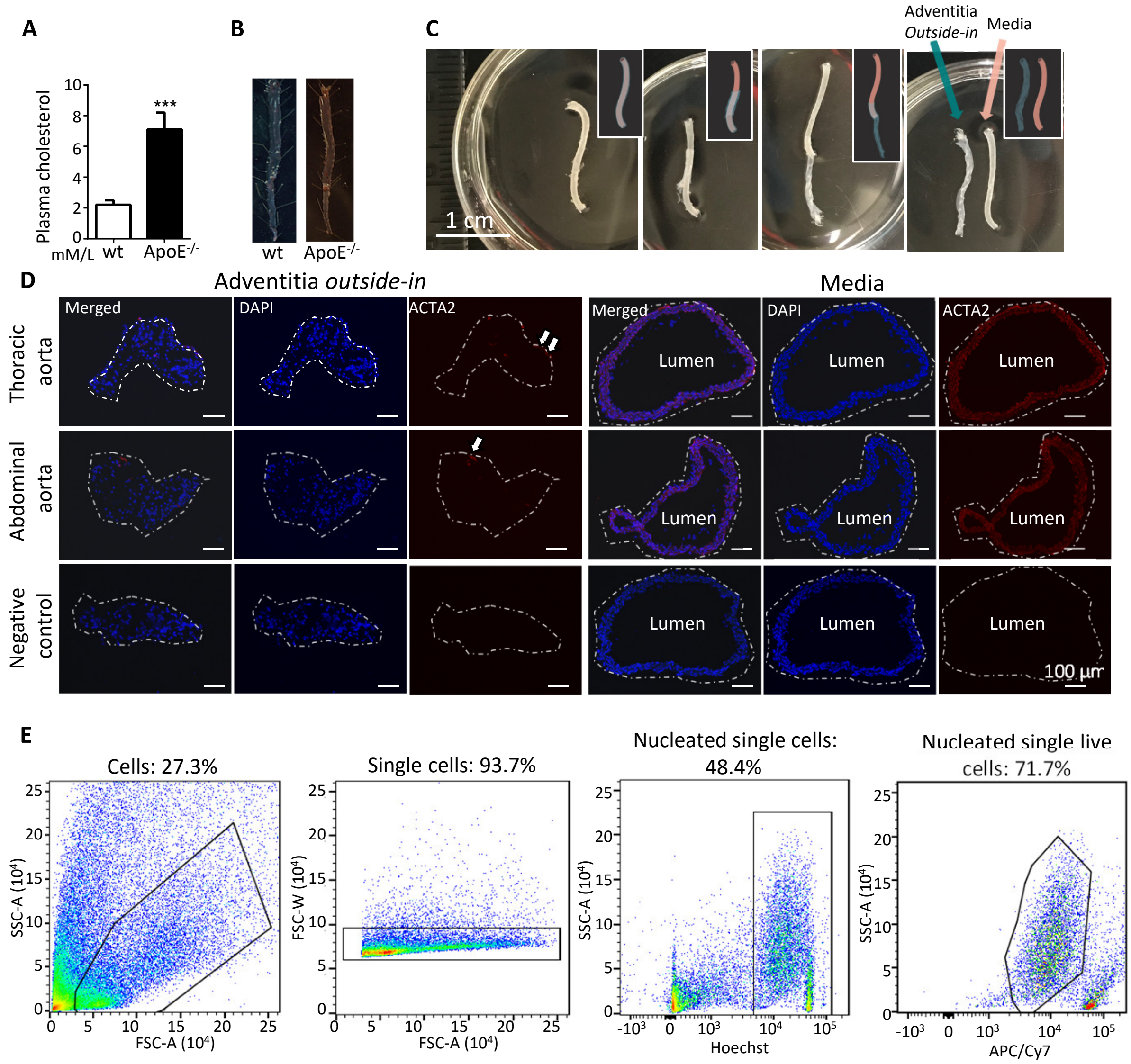

Supplementary Figure I. Phenotype of ApoE$^{-/-}$ mice and cell preparation process from aortic adventitia. (A) Blood cholesterol level of 12-week-old wt and ApoE$^{-/-}$ mice. (B) *En face* Oil red O staining of aorta from wt and ApoE$^{-/-}$ mice. (C) Graph defining the of the boundaries of adventitia taken for scRNAseq. Sketches on the top right of the image show the peeling-off process of adventitia. Only thoracic artery shown for demonstration purposes. Orange sketch and arrow indicates the media, and green sketch and arrow indicate the adventitia. (D) Staining of peeled off adventitia (in an outside-in form) and media with ACTA2. Nucleus was counterstained with DAPI. Dashed line indicates the *in vivo* contact point of media and adventitia. White arrows indicate the ACTA2 positive SMCs peeled off from the medial layer. (E) Sorting gate of single cells for RNA-sequencing. Wt, wild-type. ***P < 0.001.

A

Parameter	wt	ApoE $^{-/-}$
Estimated Number of Cells	2,271	3,153
Fraction Reads in Cells	86.50%	82.50%
Mean Reads per Cell	285,239	193,675
Fraction of Reads kept after aggregation	65.8%	100.0%
Mean Reads per Cell after aggregation	187687	193675
Median Genes per Cell	1,140	1,048
Valid Barcodes	97.00%	97.20%
Sequencing Saturation	96.40%	94.40%
Q30 Bases in Barcode	96.30%	96.30%
Q30 Bases in RNA Read	89.70%	89.80%
Q30 Bases in UMI	96.10%	96.20%
Reads Mapped Confidently to Transcriptome	67.60%	68.50%
Reads Mapped Confidently to Exonic Regions	70.50%	71.50%
Reads Mapped Confidently to Intronic Regions	12.80%	11.40%
Reads Mapped Confidently to Intergenic Regions	3.40%	3.40%

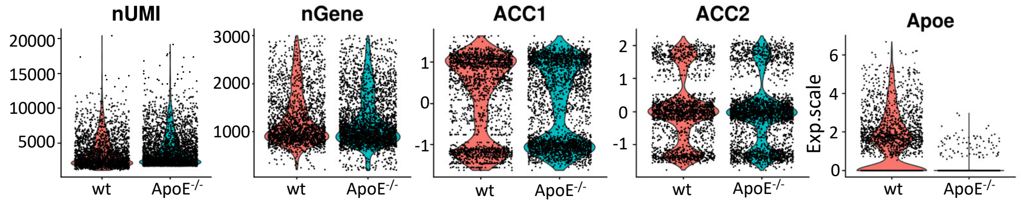

D

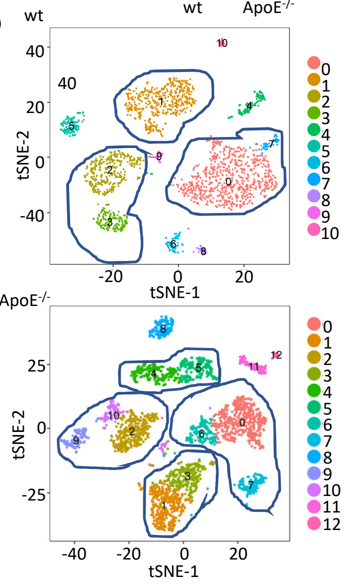

wt	Separate clustering		Integrated clustering			
Cells	Cluster	Number	Cluster	Number	Common cells	Percentage
Non-immune	2,3	485	3, 5, 15	516	470	0.910853
T and ILC	1	480	2, 12, 13	466	465	0.997854
B	0,7	828	1, 10 ,11	837	816	0.97491
Mono-Mϕ	4, 6, 8	239	4, 7, 8 ,14	249	227	0.911647
NK	5	111	6	101	99	0.980198
Eryth	9,10	73	9	43	34	0.790698

ApoE $^{-/-}$	Separate clustering		Integrated clustering			
Cells	Cluster	Number	Cluster	Number	Common cells	Percentage
Non-immune	4, 5	470	3, 5, 15	559	470	0.840787
T and ILC	2, 9, 10	639	2, 12, 13	617	605	0.980551
B	1, 3	652	1, 10, 11	716	646	0.902235
Mono-Mϕ	0, 6, 7	860	4, 7 ,8 ,14	879	841	0.956769
NK	8	150	6	150	145	0.966667
Eryth	11,12	148	9	145	144	0.993103

Supplementary Figure II. Quality of single-cell RNA-sequencing data. (A) Overall quality of scRNA-seq data. (B) Violin plots of basic features of sequencing data. nUMI, number of unique molecular identifier, nGene, number of genes, ACC1, dimension 1 of aligned canonical correlation, ACC2, dimension 2 of aligned canonical correlation. (C) Expression of *Apoe* in wt and ApoE $^{-/-}$ adventitial cells. Wt, wild-type, exp.scale, scaled expression. (D) Separate clustering of wt and ApoE $^{-/-}$ adventitia cells and their percentage (in comparison with cell number from the same type of cells of integrated dataset) of overlap cells. The major cell types are identified with markers from Fig.1G.

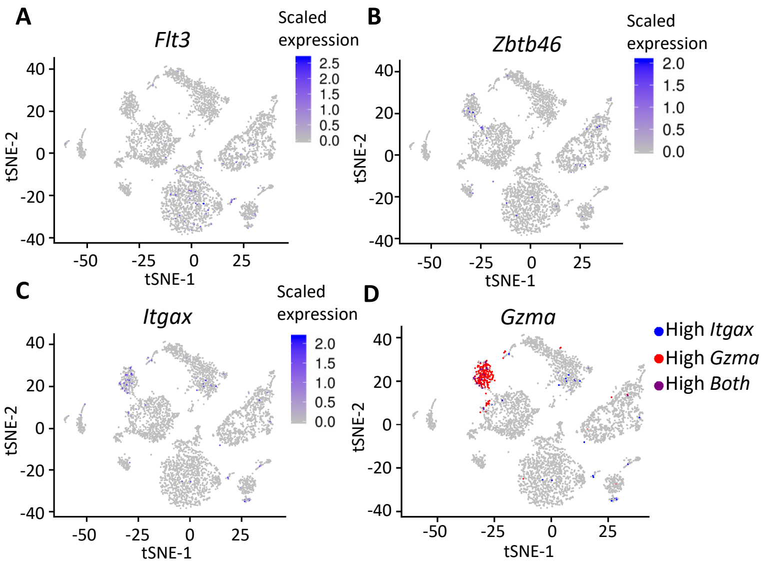

Supplementary Figure III.(A-C) Feature plot of dendritic cell markers. (D) Feature plot overlay of *Itgax* (encoding CD11c) and *Gzma* showing the co-expression of these two markers.

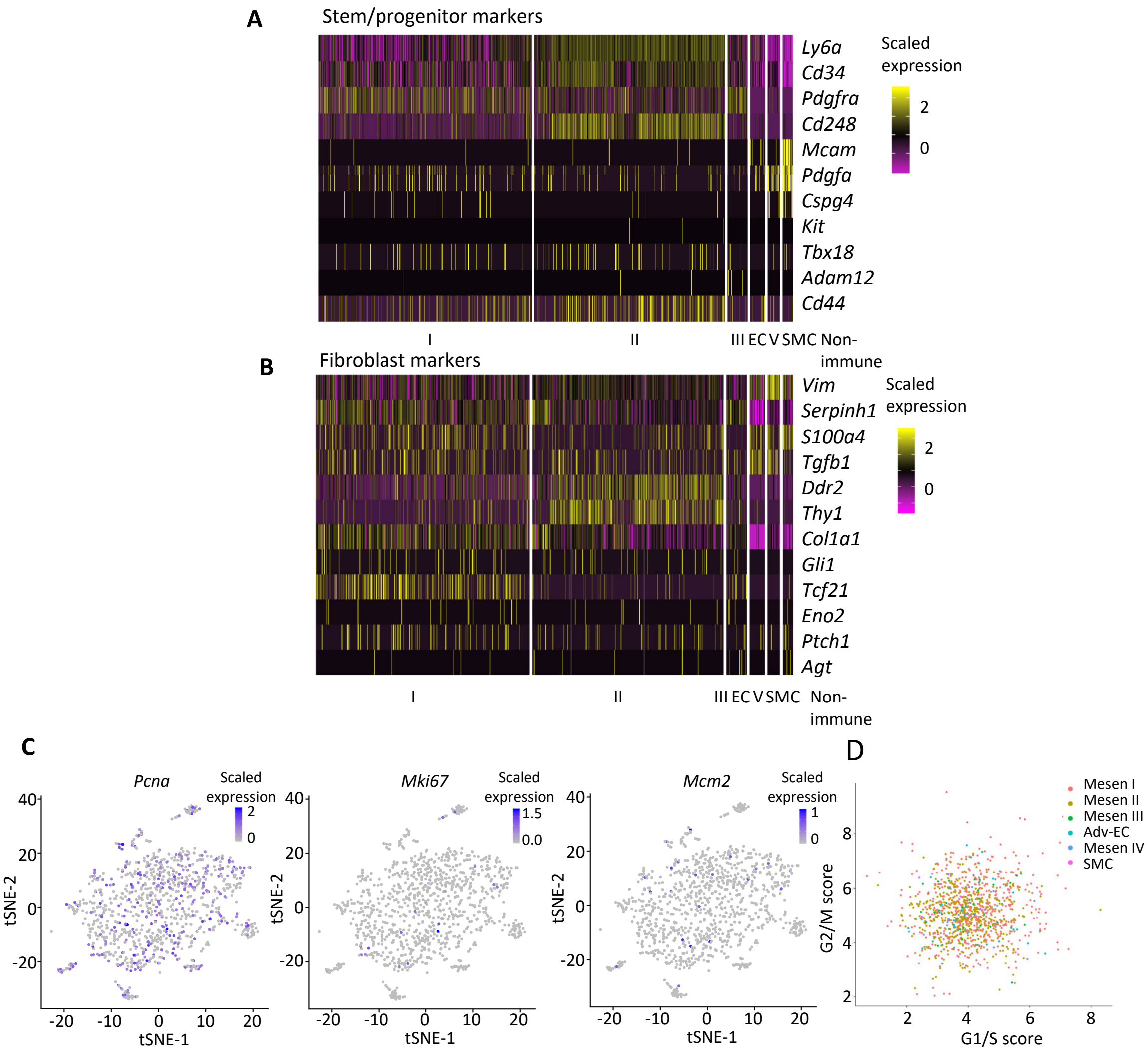

Supplementary Figure IV. (A) Heatmap of the mesenchyme stem/progenitor markers from each non-immune cluster. (B) Heatmap of fibroblast markers from each non-immune cluster. I-IV indicates Mesen I to Mesen IV clusters. (C) Feature plots of proliferation markers in the non-immune population. (D) Cell cycle analysis of the non-immune population.

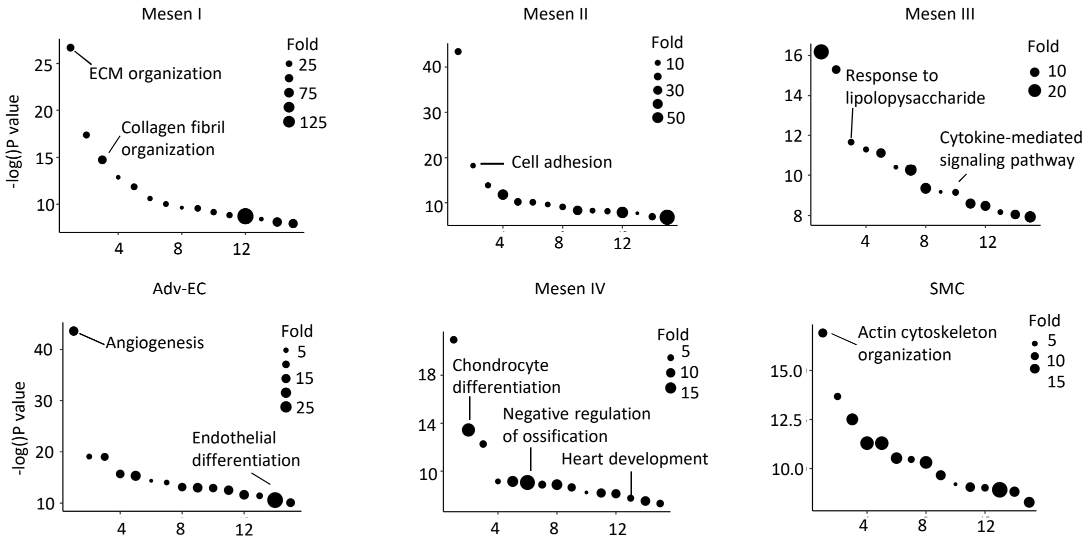

Supplementary Figure V. GO terms (biological function) analysis with marker genes of each non-immune cluster. ECM, extracellular matrix.

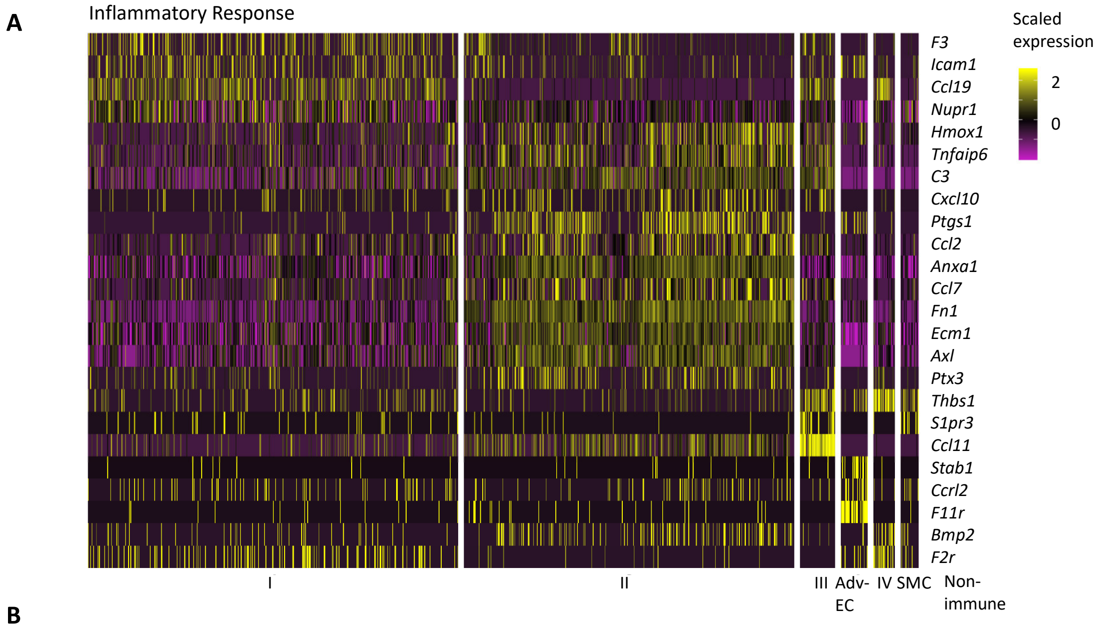

B

Non-immune cells	Cluster	Fig.2C		Fig.2D		Fig.2E	
1	Mesen I	*Mfap4*	*Igfbp7*	*Adamtsl2*	*Gas6*	*Mfap4*	*Pdgfrl*
2	Mesen II	*Ccl2*	*Ly6a*	*Pi16*	*Pla1a*	*Cd248*	*Procr*
3	Mesen III	*Ccl11*	*Lpl*	*Col5a1*	*Col4a1*	*Sparc1*	*Tmem176b*
4	Adv-EC	*Icam2*	*Tm4sf1*	*Pecam1*	*Cldn5*	*Egfl7*	*Esam*
5	Mesen IV	*Dkk3*	*Tbx20*	*Prelp*	*Wif1*	*Wif1*	*Ptn*
6	SMC	*Flna*	*Myh11*	*Tpm2*	*Acta2*	*Lmod1*	*Snca*

Supplementary Figure VI. (A) Heatmap of the markers of each cluster that are also from GO term "Inflammatory response" from each non-immune cluster. I-IV indicates Mesen I to Mesen IV clusters. (B) Markers for each non-immune cluster mentioned in the main text.

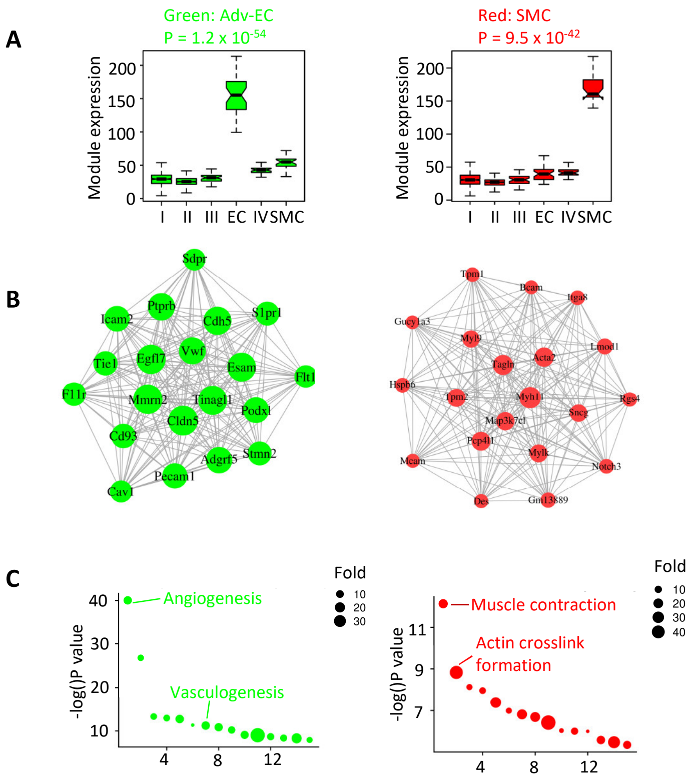

Supplementary Figure VII. Gene expression dynamics of the non-immune population. (A) Gene expression distribution of genes from the green and red module in wt and ApoE$^{-/-}$ non-immune cells were shown by boxplot. (B) Correlation network of the top 20 (by decreasing gene-module membership) genes in green and red module. (C) GO terms (biological function) analysis of green and red module genes. GOBP, gene ontology biological function.

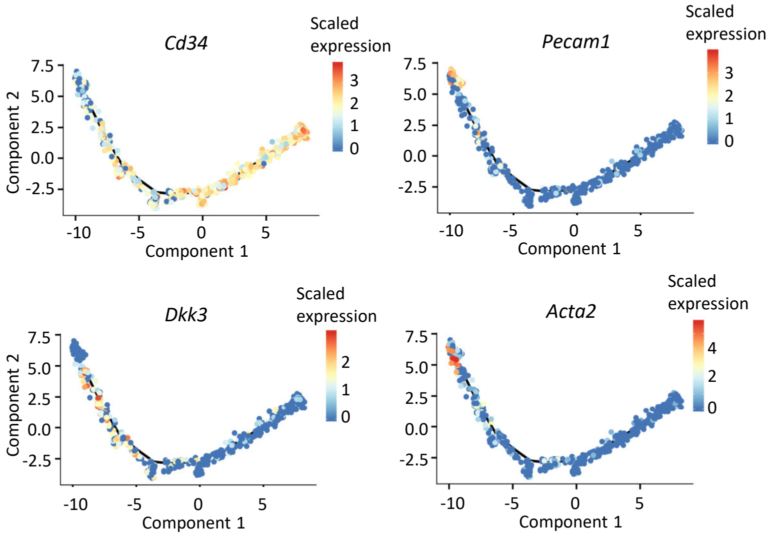

Supplementary Figure VIII. Expression level of selected marker genes (*Cd34*: Mesen II, *Pecam1:* Adv-ECs, *Dkk3 and Acta2*: SMCs) along the pseudotime trajectory.

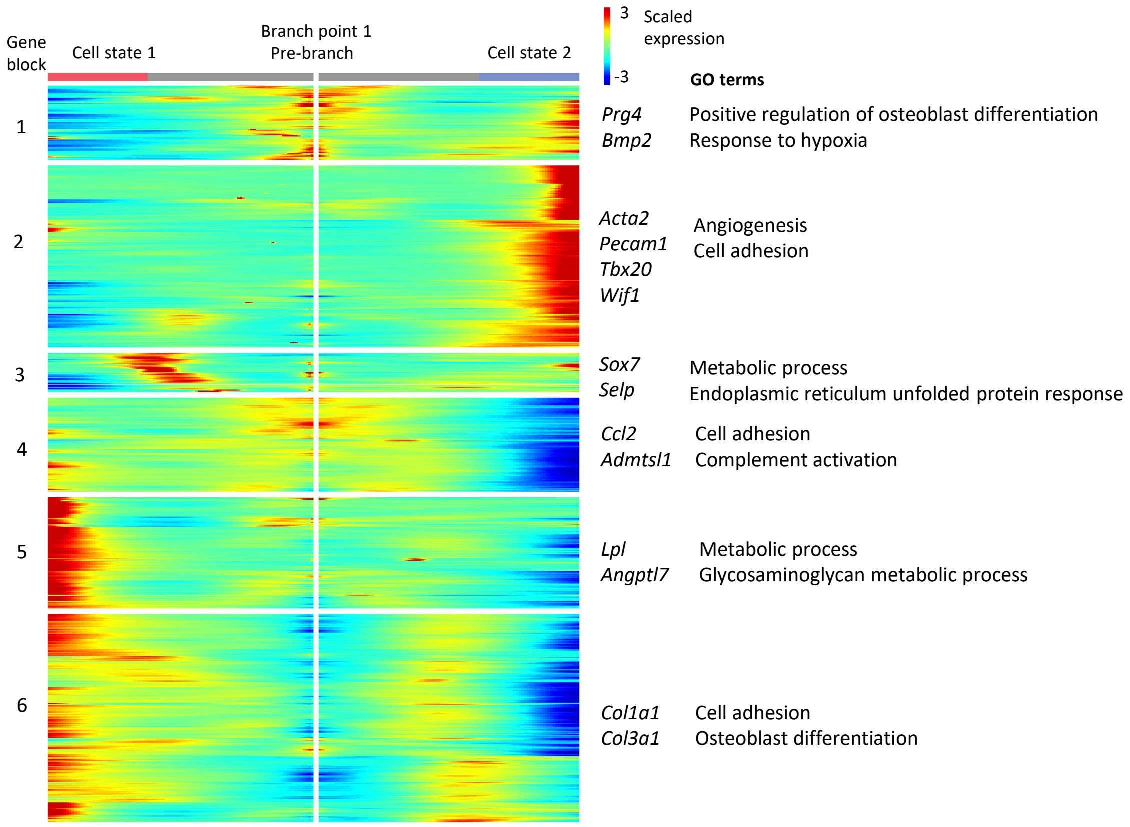

Supplementary Figure IX. Heatmap of the significantly changed genes ($P < 0.01$) discovered by the "BEAM" function from monocle in branch point 1. Selected genes and first two (by P value) GO term (biological function) from each gene cluster were shown.

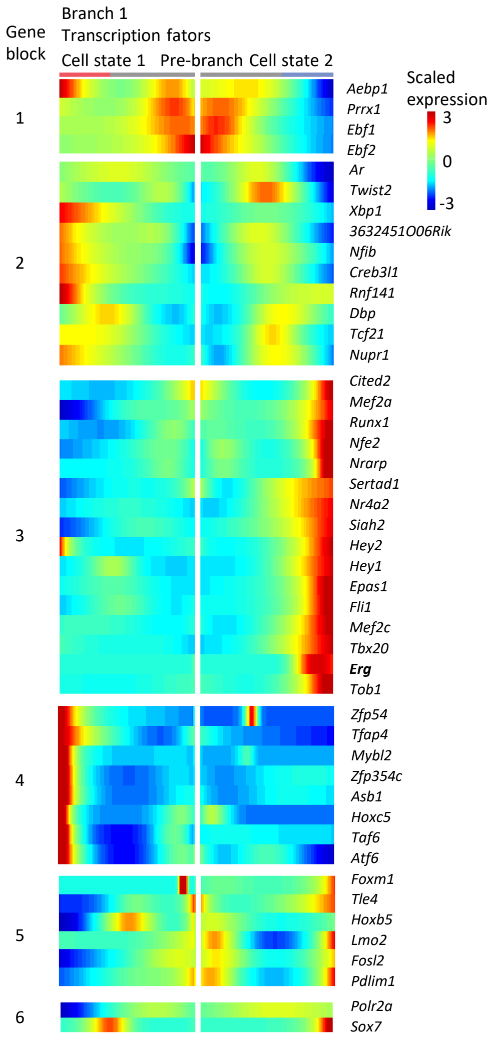

Supplementary Figure X. Heatmap of the significantly changed transcription factors ($P < 0.01$) for branch point 1.

A

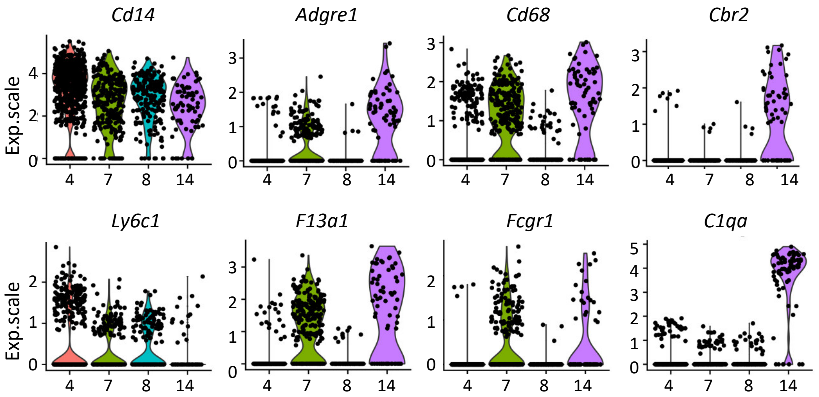

B

Mono-Mϕ Clusters		Fig.1D and 1E		Fig. 1F		Fig.5A		Fig.5B		Supp Fig. XI
4	Monocytes	***Cd14***	***Cebpb***	*Il1b*	*Cd14*	*Clec4d*	*Xcl2*	*Il1b*	*Il1r2*	
7	Inflammatory Mϕ	***Cd14***	***Cebpb***	*Ccl9*	*Hmox1*	*Ms4a6c*	*Gngt2*	*Lgals3*	*Tgfb1*	***Adgre1 F13a1 Cd68 Fcgr1***
8	Monocytes	***Cd14***	***Cebpb***	*Ly6g*	*Mmp9*	*Anxa1*	*Wfdc21*	*Ifitm6*	*Adpgk*	
14	Resident Mϕ	***Cd14***	***Cebpb***	*Ccl2*	*Pf4*	*Folr2*	*Pf4*	*Sepp1*	*Mrc1*	***Adgre1 F13a1 Cd68 Fcgr1*** *Cbr2 C1qa*

Supplementary Figure XI. (A) Violin plots of selected markers of the monocyte-macrophages showing their gene expression level. (B) Summary of markers for monocyte-macrophage clusters. Exp.scale, scaled expression. Mono-Mϕ, monocyte-macrophages.

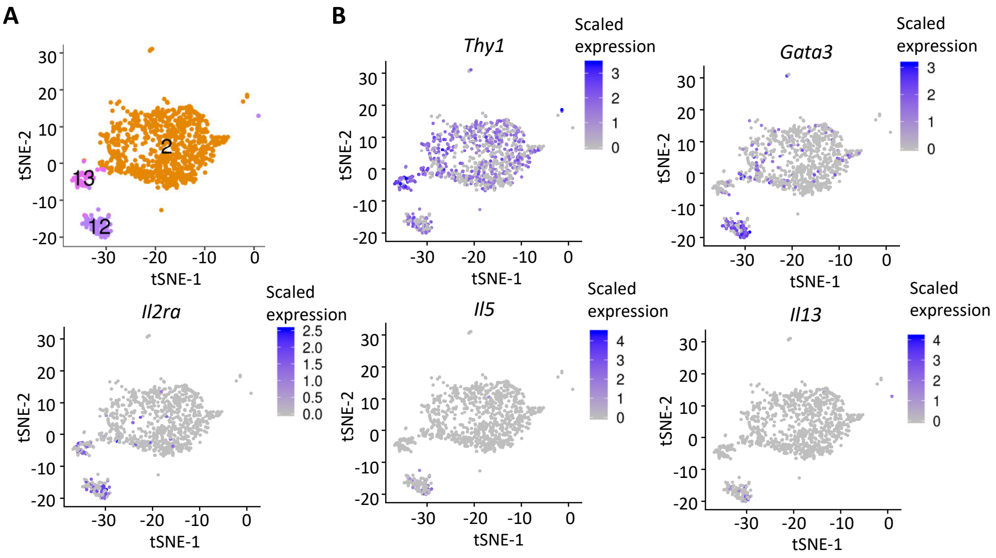

C

T and ILC	Clusters	Fig.1D and 1E	Fig.1F		Fig.5F		Fig.5G			Supp Fig. XII		
2	Mixed T	*Cd3d*	*Cd3d*	*Cd28*	*Gramd3*	*Cd2*	*Klf2*	*Lef1*	*Cd8a*			
12	ILC2	*Il1rl1*	*Il1rl1*	*Gata3*	*Arg1*	*Lmna*	*Il1rl1*	*Gata3*	*Furin*	*Il2ra*	*Il5*	*Il13*
13	Th17	*Cd3d*	*Il17a*	*Thy1*	*Cd163l1*	*Tmem176b*	*Il17a*	*Cxcr6*	*Tmem176a*			

Supplementary Figure XII. (A) TSNE plot of T lymphocytes and ICL2 cells (Cl 2, 12 and 13). Colors denote cluster identities. (B) Feature plot showing the distribution and expression level of selected genes. tSNE, t-stochastic nearest neighbor embedding, ILC2, type 2 innate lymphoid cells. (C) Summary of markers for T and ILC clusters. ILC, type 2 innate lymphoid cells, Th17, T-helper cell 17.

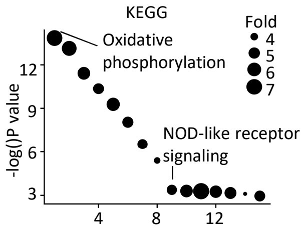

Supplementary Figure XIII. KEGG analysis of upregulated genes (log(fold change) > 0.25) in *Il1rl1* positive innate lymphoid cells from ApoE$^{-/-}$ adventitia in comparison with corresponding wt cells.

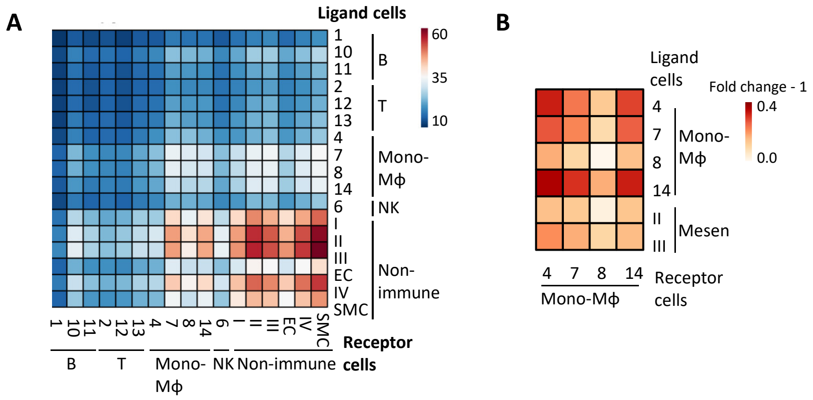

Supplementary Figure XIV. Mean interaction numbers between cell types from wt adventitia. Rows represent ligand cells and columns represent receptor cells. (B) Heatmap showing the change (fold change - 1) mean ligand-receptor interaction numbers in ApoE$^{-/-}$ adventitia in comparison with wt adventitia.

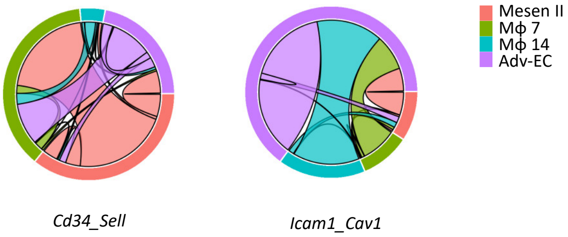

Supplementary Figure XV. Computationally obtained interaction of *Cd34* and its receptor *Sell*, *Icam1* and its receptor *Cav1* between ApoE$^{-/-}$ Mesen II, inflammatory macrophages (Mϕ 7), resident macrophages (Mϕ 14) and ECs. The same color of link and the band indicates that cells from this cluster contribute to the interaction as ligand. The different color of link and the band indicates that cells from this cluster contribute to the interaction as receptor. Same band color at both ends of the link illustrates interaction within this cell type. The band length ratio between each color and all colors represents contribution of a specific cell type to ligand-receptor interactions. Within band of a specific color, ratio between the band length with same color as the link and the total band length of this color represents contribution of a specific cell type as ligand. Within band of a specific color, ratio between the band length with different color from the link and the total band length of this color represents contribution of a specific cell type as ligand.

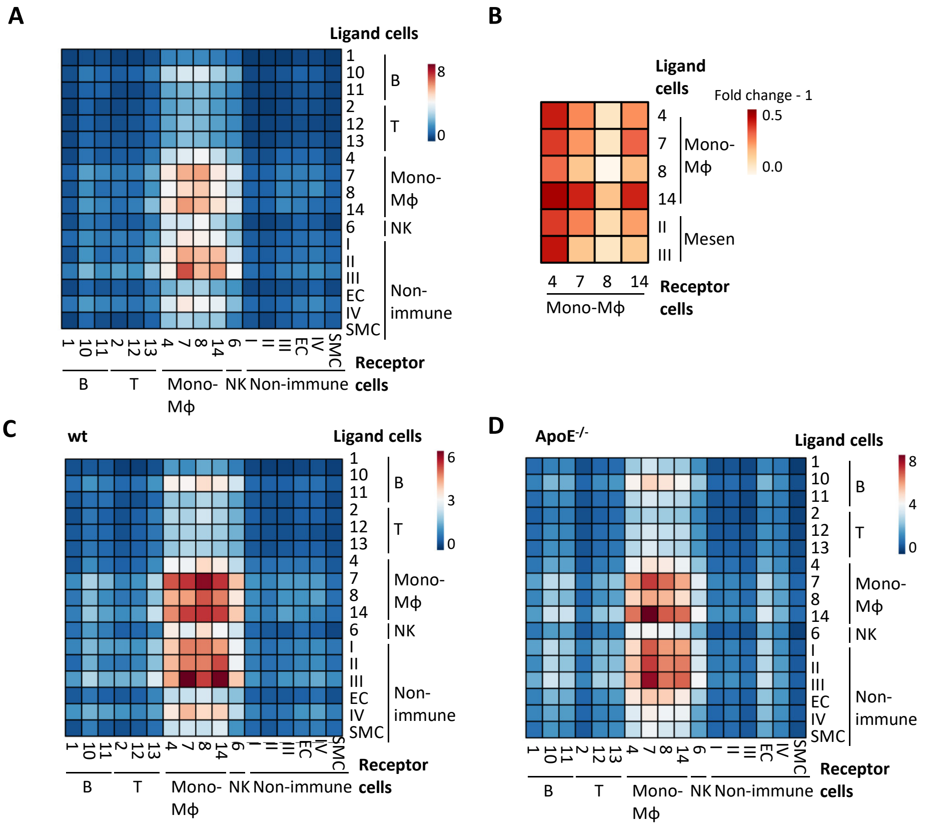

Supplementary Figure XVI. (A) Mean interaction numbers of ligands and receptors from the GO inflammatory response gene set between cell types from wt adventitia. Rows represent ligand cells and columns represent receptor cells. (B) Heatmap showing the change (fold change - 1) mean ligand-receptor (from GO inflammatory response gene set) interaction numbers in ApoE$^{-/-}$ adventitia in comparison with wt adventitia. (C and D) Re-analysis of Mean interaction numbers of ligands and receptors from the GO inflammatory response gene set between cell types from wt and ApoE$^{-/-}$ adventitia. Rows represent ligand cells and columns represent receptor cells. To avoid the influence of cell number in each cell type, in cell types with more than 100 cells, 30 cells were randomly selected for downstream analysis.

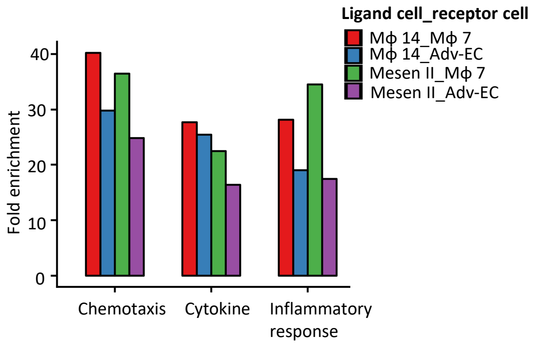

Supplementary Figure XVII. Gene set enrichment analysis (UniProtKB Keywords) of the ligands from the top 200 (mean number of interaction) ligand-receptor pairs of ligand cell type (resident Mϕ 14 and Mesen II) and receptor cell type (inflammatory Mϕ 7 and Adv-ECs). Adv-ECs, adventitia endothelial cells.

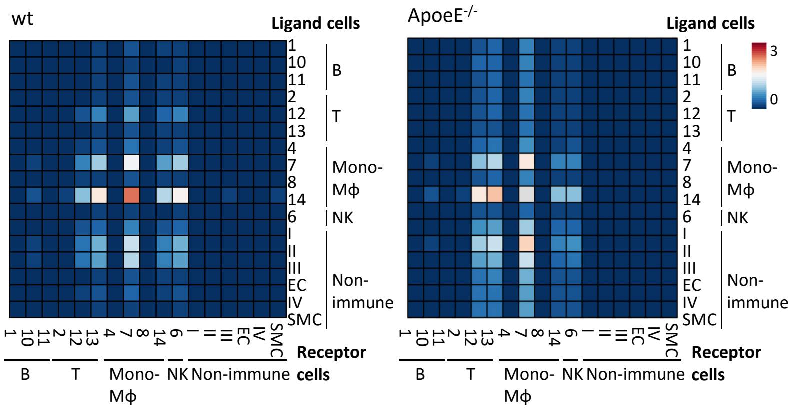

Supplementary Figure XVIII. Mean interaction numbers of *Ccl2* and its receptor *Ccr2* between cell types from wt and ApoeE$^{-/-}$ adventitia. Rows represent ligand cells and columns represent receptor cells.

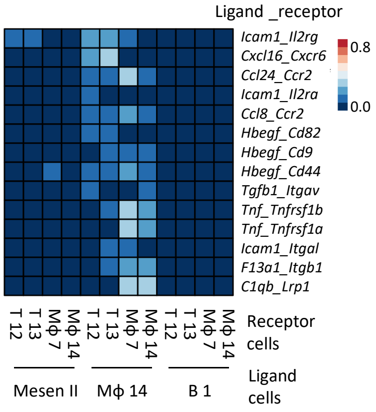

Supplementary Figure XIX. Heatmap of mean interaction numbers of specified ligand-receptor pairs between specified cell types.

A

B

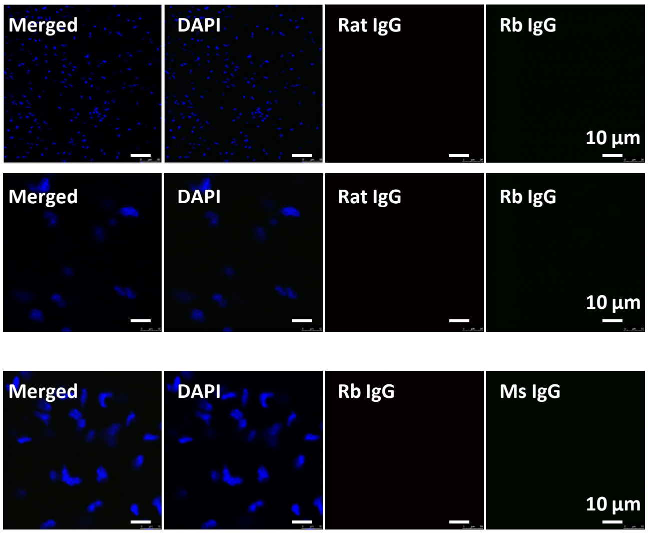

Supplementary Figure XX. (A) Negative immunostaining using IgG controls for adventitial staining with PECAM1 (Rat) and LYVE1 (Rb). (B) Negative immunostaining using IgG controls for adventitial staining with ROFOX3 (Rb) and ACHE (Ms).
