# Supplementary figures and images for: Adventitial Cell Atlas of wt (Wild Type) and ApoE (Apolipoprotein E)-Deficient Mice Defined by Single-Cell RNA Sequencing
Source: Arterioscler Thromb Vasc Biol. 2019 Apr 4;39(6):1055–71. doi: 10.1161/ATVBAHA.119.312399 (PMC6553510; doi:10.1161/ATVBAHA.119.312399)

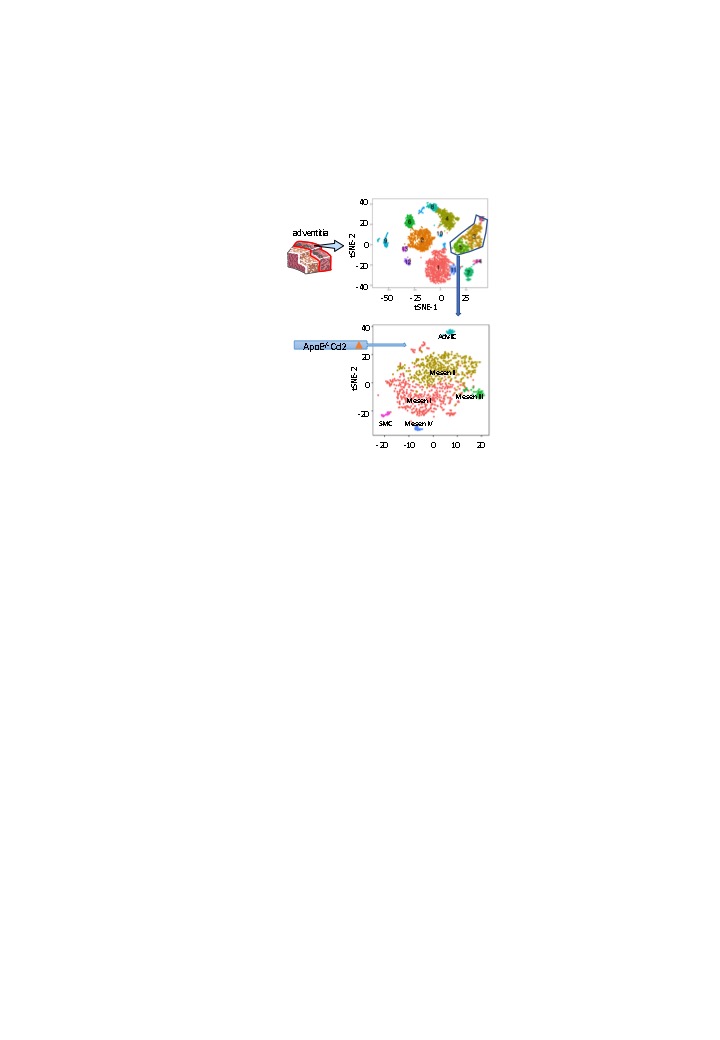

Supplement: Supplementary file 6 [file atv-39-1055-s006.jpg]
