# Supplementary material for: Adventitial Cell Atlas of wt (Wild Type) and ApoE (Apolipoprotein E)-Deficient Mice Defined by Single-Cell RNA Sequencing
Source: Arterioscler Thromb Vasc Biol. 2019 Apr 4;39(6):1055–71. doi: 10.1161/ATVBAHA.119.312399 (PMC6553510; doi:10.1161/ATVBAHA.119.312399)
Supplement: Supplementary file 7 [file atv-39-1055-s007.pdf]

Major Resources Tables

Animals (in vivo studies)

Species	Vendor or Source	Background Strain	Sex
Mouse (wt)	Beijing Vital River Laboratory Animal Technology Co., Ltd	C57BL/6	Male
Mouse (ApoE$^{-/-}$)	Beijing Vital River Laboratory Animal Technology Co., Ltd	C57BL/6	Male

Antibodies

Target antigen	Vendor or Source	Catalog #	Working dilution	Lot # (preferred but not required)
anti-LYVE1	Abcam	ab14917 (Rb)	10 μg/ml	
anti-PECAM1	BD Biosciences	553370 (Rat)	5 μg/ml	
anti-RBFOX3	Abcam	ab177487 (Rb)	17.89-8.36 μg/ml	
anti-ACHE	Life-Tech	MA3-042 (Ms)	10 μg/ml	
